# Regioisomeric Quasiracemates – Fluorinated Diarylamide, Naphthylamide, and Benzoyl Phenylalanine Systems

**DOI:** 10.1021/acs.cgd.5c00550

**Published:** 2025-07-16

**Authors:** Mu Mu W. Dun, Michaela N. Gao, Ethan C. Vyhmeister, Kraig A. Wheeler

**Affiliations:** Department of Chemistry, 7448 Whitworth University, 300 W Hawthorne Rd, Spokane, Washington 99251, United States

## Abstract

Structural families of quasiracemic materials were studied to understand how the pairwise assembly of regioisomeric quasienantiomers affects the molecular recognition process. These systems combine all variations of monofluorinated aryl groups (*i.e*., 2-/3-, 2-/4-, and 3-/4-) attached to diarylamide, naphthylamide, and benzoyl phenylalanine molecular frameworks. Including all racemates and H/F quasiracemic materials in this study emphasizes the structural trends and cocrystallization complexities required to generate positional quasiracemates. This work is supported by crystallographic assessments, crystal lattice energy calculations, and video-assisted hot stage thermomicroscopy.

## Graphical abstract

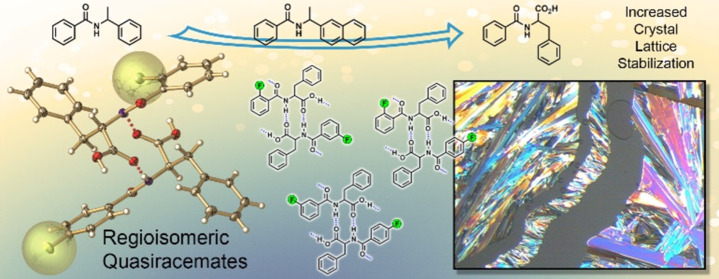

## Introduction

The field of quasiracemic materials[Fn fn1] has experienced considerable development since the early days of Pasteur, when, in the 1850s, quasiracemates were described as combination isomers likely created using locally sourced chemical feedstocks – i.e., malic acid (from apple orchards) and tartaric acid (from the wine industry).^1^ Building on Pasteur’s seminal work, several entries in this field occurred in the early part of the 20th century that demonstrated how the quasiracemate approach could be utilized to advantage in probing the steric properties and stereochemical relationships of near enantiomers.^2–4^ This combined effort examined Pasteur’s systems and others by assessing the optical and thermal material properties. The hallmark of many of these studies was the creation of detailed melting-point phase diagrams, a technique that would become the method of choice for examining quasiracemic systems for many decades. Influenced by this early work, Fredga and co-workers continued these efforts by investigating racemic and quasiracemic systems[Fn fn1] to understand the spatial correlations of optically active materials and their biofunction as plant growth regulators. Given Fredga’s five decades of contributions to the field from the 1930s to the 1980s, including two reviews,^5,6^ it is no surprise that Fredga’s work is generally credited with developing quasiracemates into a mature discipline. Several important themes emerge when considering the results of this early work. The design strategies of quasiracemate studies lack any noticeable molecular framework preference, and experiments from this era suggest that using a single point of structural variance is best. Additionally, when considering the pairwise selection of quasienantiomers, the imposed structural differences should consist of, at most, modest changes of the type −CH_2_–, −O–, −S–, −Se– ^7–13^ or −CH_3_, −Cl, −Br, −NO_2_, −OCH_3_, I.^14–21^ Pairing quasiracemic components using these entries is largely expected, given their similar topological properties.

Equally telling and no less critical to the development of the quasiracemate approach are systems that receive less attention or failed examples of quasiracemic cocrystallization. One such area is the use of positional isomers, also known as regioisomers, to generate quasiracemic materials. Regioisomers – compounds with identical molecular formulas that differ by chemical connectivity represent a general class of compounds that have experienced limited attention in the quasiracemate literature. Two such reports show the success of using the 2,4-dichloro-/3,4-dichlorophenylsulfinylacetic acid^22^ and *n*-butyl-/*i*-butylsuccinic acid^23^ quasiracemates. Other accounts with regioisomers describe failed attempts using the 3-iodo-/4-iodophenoxypropionic acid^24^ and α-(1-naphthyl)-/α-(2-naphthyl)­propionic acid^25^ systems. Reports of quasiracemates beyond the 1980s lack examples of regioisomers. The scarcity of instances highlighting regioisomeric quasiracemates, both successes and failures, are all the more intriguing, given that these isomeric compounds were readily available and prepared during the same time frame and at the same research site. The historical literature supports the notion that practitioners in the field were either hesitant to pursue regioisomeric quasiracemates or report their failed attempts. Nonetheless, a survey of the literature suggests that such building blocks are incompatible with the quasiracemate approach. Our limited efforts with regioisomeric quasiracemates validate these classes of materials as largely unsuitable for the pairwise assembly of small molecule quasienantiomers.

This report systematically examines quasiracemate behavior by assembling regioisomeric quasienantiomers using three related molecular architectures – *i.e*., diarylamide **1**, naphthylamide **2**, and benzoyl phenylalanine **3** (Scheme 1). These systems have recently received attention as quasiracemate templates and are included in this study to understand how variations in crystal lattice organization and stabilization affect molecular recognition processes. Racemates and quasiracemates of **1** – **3** constructed as the 2-, 3-, and 4-substituted fluorobenzoyl isomers were prepared and assessed using single-crystal X-ray crystallography, crystal lattice energy calculations, and hot stage thermomicroscopy.

**Scheme 1:**
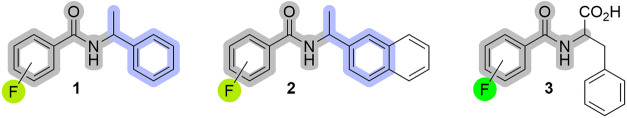
Diarylamide (**1**), Naphthylamide (**2**), and Benzoyl Phenylalanine (**3**) Molecular Frameworks for Quasiracemate Studies^a^

a Highlighted fragments indicate structural similarities.

The selection of fluoro-substituted derivatives targeted in this report relied on our previous experience with systems **1** and **2**, where more spatially distinct substitutions, such as H and Cl, resulted in unsuccessful quasiracemic cocrystallizations.^26–28^ Additionally, these same early efforts also explored fluoro-functionalized materials, showing the potential utility of these compounds for probing the structural boundary of positional quasiracemates.

## Experimental Section

### Synthetic Procedure

All chemicals and solvents were purchased from VWR Scientific or MilliporeSigma and used as received without further purification. The synthetic strategies to prepare the diarylamide **1**,^26,27^ naphthylamide **2**,^28^ and benzoyl phenylalanine **3** ^29^ derivatives were taken from previous reports. See the Supporting Information for reaction yields and NMR assignments.

### Crystal Growth Methods

Suitable samples for X-ray diffraction experiments involved slow evaporation under ambient conditions using methanolic solutions. This process combined 10–20 mg of the *S* and *R* racemic and quasiracemic components in a 1:1 molar ratio, with crystals forming in 1–4 days. Multiple recrystallization attempts were often necessary, especially in the case of **1** and **2**, to achieve X-ray quality samples. Despite these efforts, several of the targeted crystal growth experiments were unsuccessful for **1**-2F/**1**-3F, (±)-**2**-2F, **2**-H/**2**-2F, **2**-H/**2**-3F, **2**-2F/**2**-3F, **2**-2F/**2**-4F, and **2**-3F/**2**-4F.

### Crystallography

Single-crystal diffraction data were collected at 100 K using a Bruker D8 Venture IμS microfocus dual-source diffractometer equipped with a PHOTON II CPAD detector and an Oxford cryogenic 800 system. Monochromatic Cu Kα (λ = 1.54178 Å) or Mo Kα (λ = 0.71073 Å) radiation sources were used to collect diffraction data using phi (φ) and omega (ω) scan strategies. Cell measurement, data collection, integration, scaling, and absorption correction were achieved using the APEX4 software.^30^ Data were processed using SAINT,^30^ and an absorption correction was performed using SADABS programs incorporated in APEX. The structure was solved using SHELXT^31^ and refined using the SHELXL^32^ program, both implemented in OLEX2.^33^ The non-hydrogen atoms were located from difference Fourier syntheses and refined with anisotropic thermal parameters. The CH hydrogen atoms were placed at calculated positions and refined using a riding model with appropriate HFIX commands and Uiso = 1.2 × Ueq (1.5 × Ueq for methyl groups). NH and OH hydrogen atoms were located in difference Fourier syntheses (NH, 1.2 × Ueqiv; OH, 1.5 × Ueqiv), and their positions refined independently. The N–H and O–H distances were restrained to 0.85(2) Å using a DFIX command when appropriate. The programs OLEX2 ^33^ and X-Seed^34^ were used for packing analysis and molecular graphics. Crystallographic data and hydrogen-bond parameters are summarized in Tables S1 and S2, respectively.

Crystallographic data for several of the targeted systems were previously reported in the CCDC Cambridge Structural Database^35^ (CCDC–CSD) and include several entries from diarylamide **1** [(±)-**1**-H (YAYHAL), (±)-**1**-3F (YAYGUE), (±)-**1**-4F (YAYHUF), (*S*)*-* **1**-H/(*R)-* **1**-2F (WANLUV), (*R*)*-* **1**-H/(*S*)-**1**-3F (YAYGEO), and (*R*)-**1**-H/(*S*)-**1**-4F (YAYGAK)] and naphthylamide **2** [(±)-**2**-H (WABJUI), (±)-**2**-4F (WABFEO), and (*S*)*-* **2**-H/(*R*)-**2**-4F (WABFAK)], and benzoyl phenylalanine **3** [(±)-**3**-H].

### Crystal Structure Similarity Searches

The degree of isomorphism with the crystal structures of **1** – **3** was quantified using the Packing Similarity Search tool provided with CCDC-Mercury (CCDC–CPSS)­(version 2024.3.1).^36^ The search parameters included clusters of 15 molecules that permitted chemical differences, inverted structures when appropriate, and included hydrogen atoms in the calculations. Output for each crystal structure pair provides information about the degree of fit via a root-mean-square (RMS) fit approach and the number of common molecules within the specified search constraints. Crystal structures modeled with disorder were processed using files consisting of whole molecules limited to one disorder component. In the case of multiple similarity search values for a given structure, the outcome with the best fit (*i.e*., largest cluster similarity and smallest RMS value) was reported in Tables 1–3.

**Table 1:**
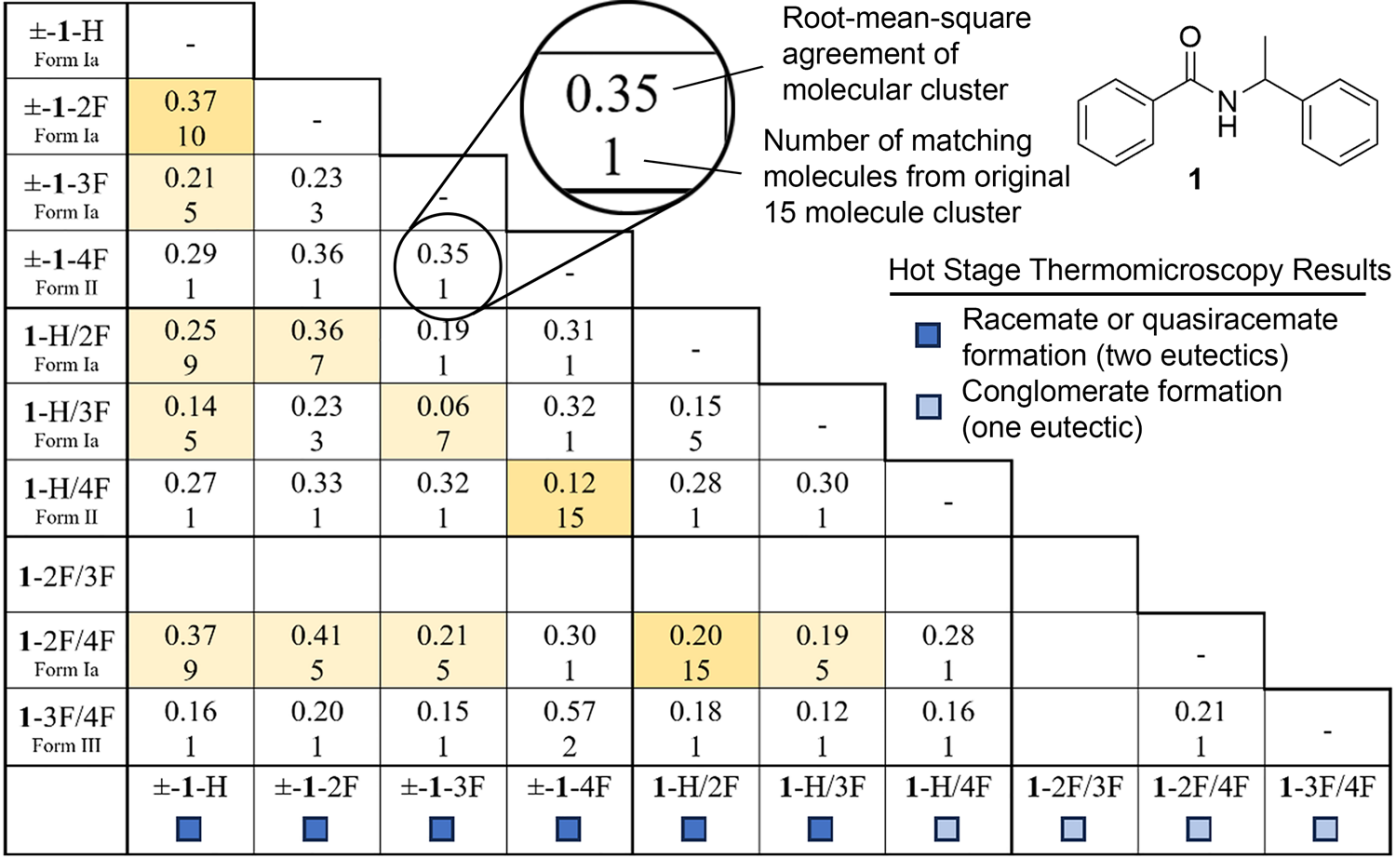
CCDC Mercury Crystal Packing Similarity Search and Hot Stage Results for Diarylamide **2**

### Crystal Lattice Energy Assessments

Crystal lattice energies (*E*_Latt_) were determined using atomic coordinates retrieved from the crystallographic data for the **1** – **3** systems. The software platform Crystal Explorer,^37^ equipped with Guassian16,^38^ was used to create molecular clusters starting from a cluster radius of 10 Å. *E*_Latt_ values were computed by direct summation of interaction energies (*i.e*., electrostatic, dispersion, polarization, and repulsion) using the central molecule and molecules included in the cluster. Several of the targeted systems exhibit disorder. In those cases, the lattice calculations were performed separately on each disorder component, and their contribution to *E*_Latt_ was weighted using the occupancy factors determined from the crystal structure refinement. Final *E*_Latt_ values for the *Z*’ > 1 crystal structures were determined by averaging the contributions from each symmetry-independent molecule.

### Video-Assisted Hot Stage Thermomicroscopy

The hot stage experiments conducted as part of this study were performed using the same instrumentation and experimental protocols as described in a previous report.^29^

## Results and Discussion

This study examines the interplay of positional isomers using three structurally related molecular scaffolds for constructing quasiracemic materials. Systems **1** and **2** differ by an aryl group, and **3** effectively increases the hydrogen-bond capacity compared to the other frameworks. Given these design elements of increased molecular size and nonbonded contacts, naphthylamide **2** and benzoyl phenylalanine **3** are expected to provide additional crystal lattice stabilization compared to diaryl **1**. This aspect of the project is critical, given our prior experience with these systems and the anticipated challenge of pairing quasienantiomeric positional isomers for many of these systems.

By cocrystallizing all possible sets of monofluorinated quasienantiomers (*i.e*., 2-/3- and 2-/4- as well as 3-/4-) and utilizing the three molecular scaffolds, this study provides significant insight into how the imposed structural variants influence the success of cocrystallizing the intended quasiracemic materials. It is important to note that by combining sets of positional quasienantiomers (*e*.*g*., **3**-2F and **3**-3F), the topological differences with the opposing H and F sites control the success of quasiracemate formation. The extent of these differences can be readily determined by comparing atomic volumes. Using the Bondi^39^ radii of 1.20 (H) and 1.47 (F) Å and atomic volumes of 7.2 (H) and 13.3 (F) Å^3^ yields a percent volume difference (%Δ*V*) of 59.5%. Although this comparison seems straightforward, the issue is further complicated when considering the range of values reported for H (1.00–1.67) and F (1.41–1.47) radii.^39–42^ Applying these values results in %Δ*V*_max_ = 95.0%^40^ and %Δ*V*_min_ = 4.5%.^41^ While there is a benefit to having a more unified set of atomic radii, it is also readily apparent from this study and other reports that the sizes of H and F and their contributions to molecular polarity and crystal packing are not interchangeable. The structural interplay between the H and F substituents is evident from the systems reported in this study and the diversity of their crystal structure patterns.

To understand the importance of the targeted F/F positional isomers to molecular recognition, this study includes racemic and H/F-substituted quasiracemic materials and their crystallographic, lattice energy, and thermomicroscopy assessments. This strategy provides a valuable approach to gaining insight into how molecular shape influences molecular assembly.

### Crystallographic Assessments

Each of the three molecular frameworks comprises ten unique compounds (*i.e.,* four racemates, three H/F quasiracemates, and three F/F quasiracemates), resulting in a total of 30 possible unique entries across the three systems. Crystal growth of these materials was unsuccessful for six entries – *i.e*., **1**-2F/**1**-3F, **2**-H/**2**-2F, **2**-H/**2**-3F, **2**-2F/**2**-3F, **2**-2F/**2**-4F, and **2**-3F/**2**-4F – despite multiple attempts using various solvent systems and recrystallization techniques. All quasiracemic crystal structures examined show components with near-inversion symmetry alignment isolated to space groups *P*1, *P*2_1_, and *C*2, mimicking the centrosymmetric *P*1̅, *P*2_1_/*c*, and *C*2/*c* groups, respectively (Figures 1–3). This structural information has been further examined for the degree of isomorphism using the Crystal Packing Similarity Search tool provided with CCDC-Mercury (version 2024.3.1) (Tables 1–3).^36^ No polymorphic phases were observed, and the presence of disorder in these structures was expected due to the similar shape and size of the hydrogen and fluorine atoms. Ten of the 25 crystal structures examined exist with crystallographic disorder. This crystallographic phenomenon includes eight entries with H/F substitutional disorder [(±)-**1**-2F, (±)-**1**-3F, **1**-H/**1**-3F, **1**-2F/**1**-4F, (±)-**2**-3F, (±)-**3**-3F, **3**-H/**3**-2F, **3**-H/**3**-3F] and four entries displaying rotational disorder of the molecular framework (**1**-H/**1**-2F, **1**-2F/**1**-4F, (±)-**2**-2F, (±)-**2**-3F). Also, despite the abundance of organic fluorine atoms in these structures, there is a lack of or no clear trend for C–H···F–C_Ar_ and C_Ar_-F···F–C_Ar_ interactions. Due to the high degree of observed isomorphism with each framework type, these nonbonded contacts likely take on secondary roles to other, more prevalent crystal packing forces. These observations are consistent with previous reports, which have shown that organic fluorine participates in various interactions, with few instances demonstrating a convincing control over molecular assemblies.^43,44^

All racemic and quasiracemic crystal structures involving molecular framework **1** assemble the components using N–H···O=C interactions into *C*4 hydrogen bond graph set motifs^45,46^ (Figure 1A). Despite this common nonbonded contact, considerable structural diversity exists within this set of structures. These crystal structures organize the components in triclinic and monoclinic space groups, where five of these entries exhibit disorder of the F and benzoyl aryl groups, and four exist with more than one symmetry-independent molecule (*Z’* = 2). Such structural variations hold importance for the general theme of the study, as they underscore how seemingly minor molecular modifications can translate into significant differences in molecular topologies and crystal packing.

**Figure 1:**
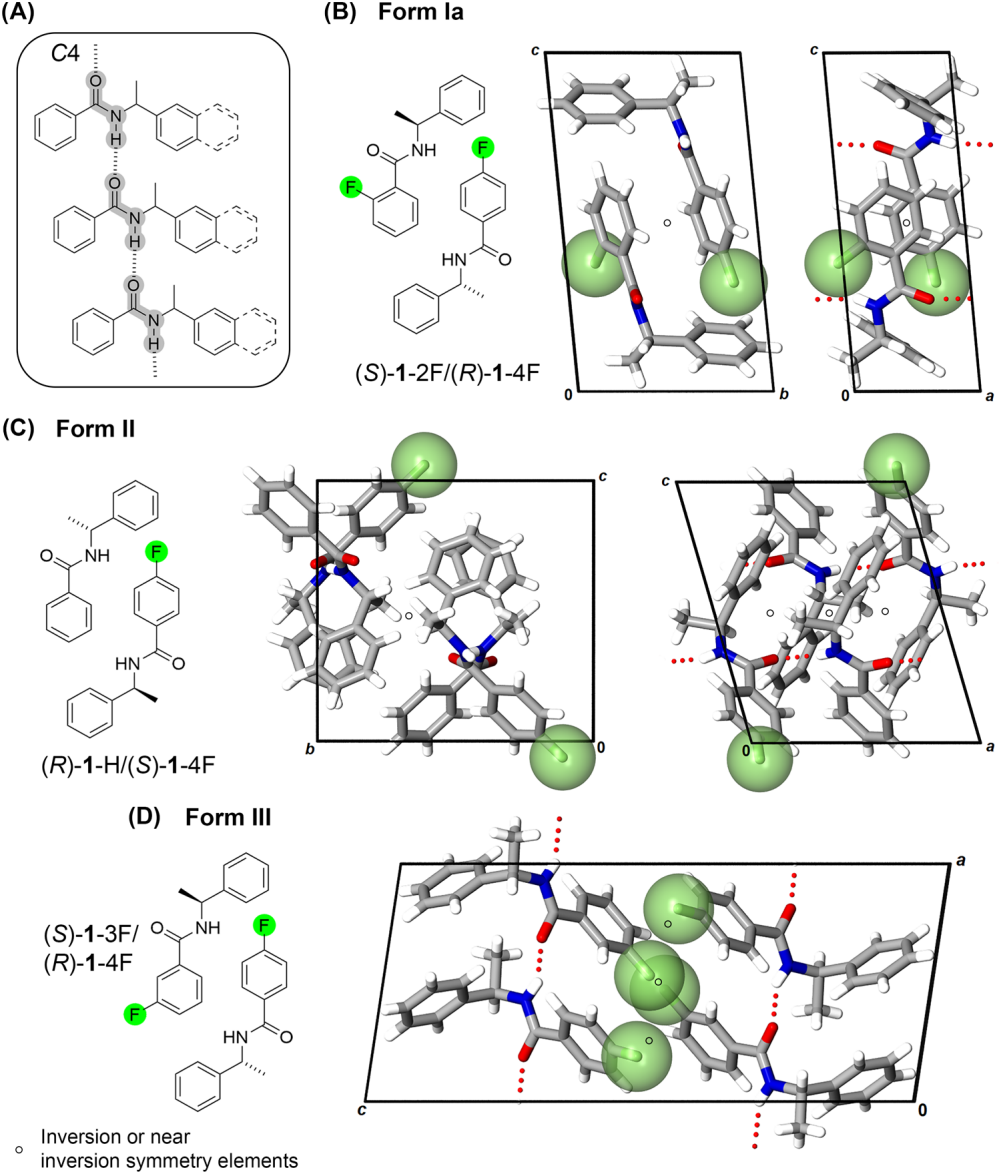
(A) Hydrogen-bond *C*4 motifs for arylamides **1** and **2**. (B–D) Examples of diarylamide **1** crystal structures showing Form Ia – III packing.

Six diarylamide **1** structures exist in Form Ia, defined by components aligned into *C*4 motifs via translationally related homomeric patterns (Figure 1A,B). These 1D motifs observed in Form Ia further organize with adjacent molecular chains via inversion (racemates – (±)-**1**-H, (±)-**1**-2F, (±)-**1**-3F) or near-inversion (quasiracemates – **1**-H/**1**-2F, **1**-H/**1**-3F, **1**-2F/**1**-4F) symmetry. Form Ia quasiracemates cocrystallize in space groups *P*1 or *P*2_1_, with molecular alignment that mimics the centrosymmetric *P*1̅ and *P*2_1_/*c* space groups found in the corresponding racemic structures. An alternative crystal packing scenario (Form II) includes similar C4 motifs, with the molecular chains constructed from 2_1_-screw-related components. Like Form Ia, the neighboring *C*4 motifs in Form II create inversion ((±)-**1**-4F) and approximate inversion (**1**-H/**1**-4F) colinear arrays; however, these adjacent chains form interdigitated interlayer regions (Figure 1C). Similar unit cell parameters and crystal packing features indicate that the crystal structure entries assigned to Form Ia and II display a high degree of isomorphism.

Form III occurs in the case of quasiracemate **1**-3F/**1**-4F with the anticipated *C*4 motifs producing distinct molecular packing due to an extensive network of H···F and F···F contacts near vdW surfaces (Figure 1D).

As shown in Table 1, applying a Crystal Packing Similarity Search (CPSS) feature provided by CCDC-Mercury^36^ to the structures with Form Ia and II offers a quantitative measure of these isomorphic relationships. Starting from a cluster of 15 molecules, the data in each cell indicates the number of matching molecules in the cluster (maximum 15) and packing similarity as a root-mean-square value. Three structure pairs show a high similarity with ≥ 10 molecules in the cluster match. Nine additional entries display a moderate relationship with 4–9 molecules in the cluster.

Cocrystallization of the racemic and quasiracemic systems of naphthylamide **2** effectively aligned the components in the crystal with the expected *C*4 motifs (Figure 2A). As previously described, systems based on **2** should offer improved crystal lattice stabilization due to the increased molecular size. Even so, this system presented considerable crystal growth challenges compared to **1**, where multiple recrystallization attempts were frequently required to achieve X-ray-quality samples.^28^ One indication of this difficulty is that only five of the ten targeted naphthylamide systems resulted in crystal structures. Omissions from this structural family curiously center on the quasiracemic systems and include two HF (**2**-H/**2**-2F and **2**-H/**2**-3F), and each of the three F/F (**2**-2F/**2**-3F, **2**-2F/**2**-4F, **2**-3F/**2**-4F) quasiracemates. The successful racemic [(±)-**2**-H, (±)-**2**-2F, (±)-**2**-3F, and (±)-**2**-4F] and quasiracemic (**2**-F/**2**-4F) crystal structures assemble the components into *C*4 translationally related chains (Form Ib), comparable to Form Ia observed with the diarylamide **1** system (Figure 2). This high degree of isomorphism is somewhat surprising given the variation of space groups for this set of structures (*i.e*., *P*1, *P*1̅, *Cc*, and *C*2/*c*).

**Figure 2:**
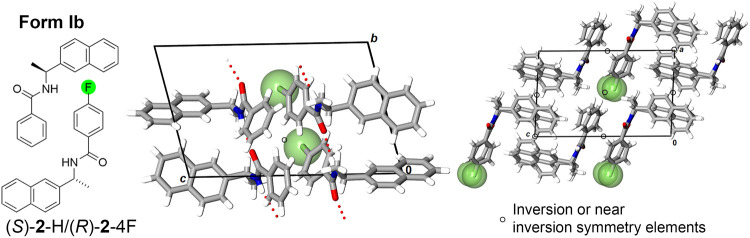
Crystal structure projections of quasiracemate (*S*)-**2**-H/(*R*)-**2**-4F showing Form Ib molecular organization.

The crystal structures of naphthylamide **2** display comparable packing patterns described for diaryamide **1**. While the study of **2** produced fewer crystal structures, applying the CCDC–CPSS tool to the structures of **2** indicated that the entries are isomorphic, with seven of the 11 pairs showing molecular clusters of ≥ 10 molecules (Table 2).

**Table 2:**
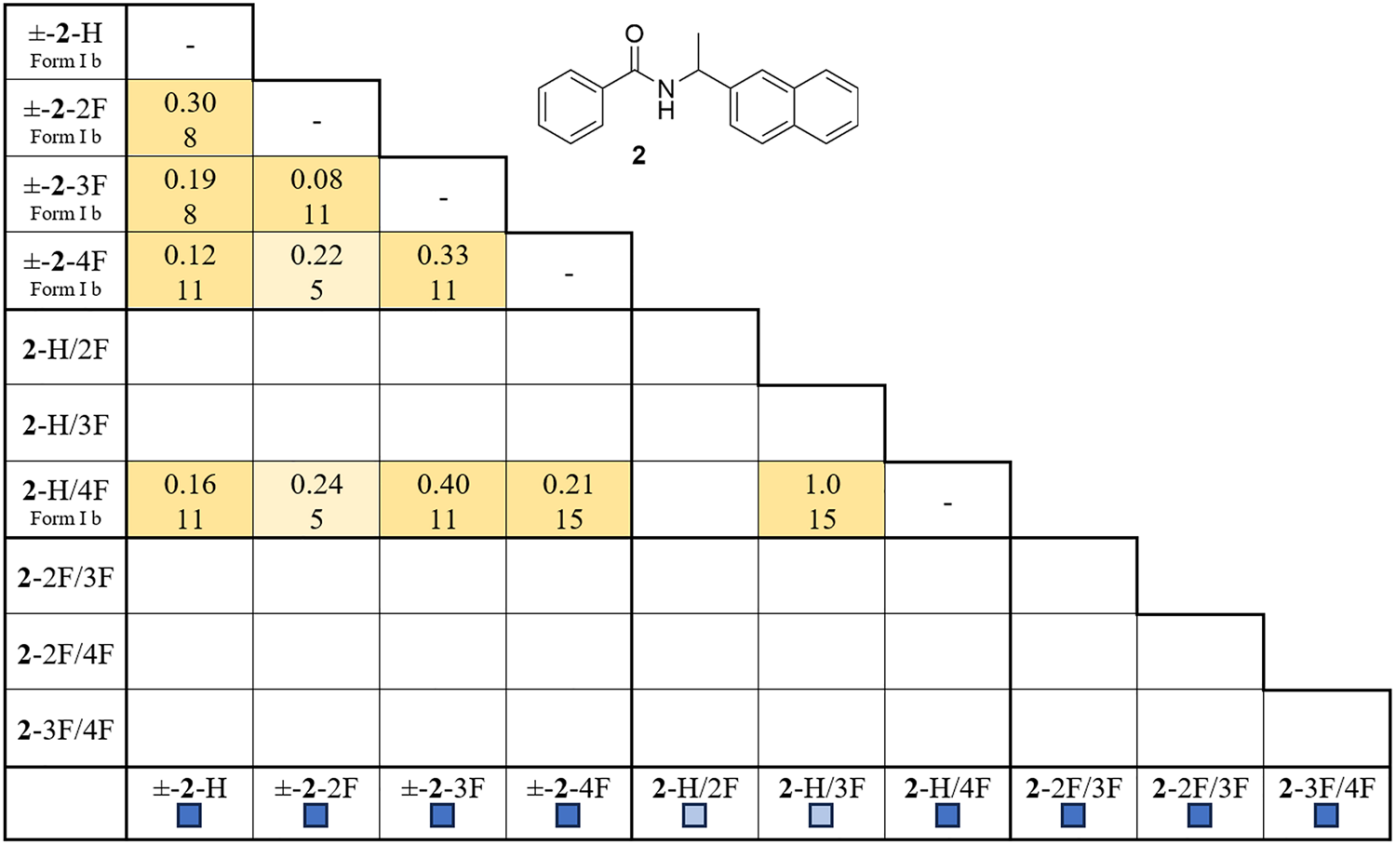
CCDC Mercury Crystal Packing Similarity Search and Hot Stage Results for Naphthylamide 2^a^

a Cell values indicate root-mean-square agreement of the structural pair (top) (maximum 15 molecules). The light and dark-shaded cells indicate entries with 3–9 and 10–15 molecules in the cluster, respectively. Outcomes from hot stage experiments are represented by dark (racemic and quasiracemic formation) and light-colored (conglomerate formation) boxes.

The design strategy for benzoyl phenylalanine **3** incorporated an additional carboxyl group into the molecular framework to investigate how increased crystal lattice stabilization impacts cocrystallization success. Due to this alteration of the molecular framework, the crystal structures of **3** exhibit entirely different crystalline landscapes compared to those of **1** and **2**. Perhaps a key observation is that, unlike 1 and 2, each of the targeted ten racemic and quasiracemic systems yields crystal structures. This improved success may be attributed to the formation of additional hydrogen bonds, where the components organize into Forms IV–VI, as seen in a recent report.^29^

Seven entries [(±)-**3**-H, (±)-**3**-4F, **3**-H/**3**-2F, **3**-H/**3**-3F, **3**-H/**3**-4F, **3**-2F/**3**-4F, **3**-3F/**3**-4F] create Form IV alignment (space groups *P*2_1_/*c* or *P*2_1_), where the N–H···O=C and N–H···O=C interactions generate *R*_2_^2^ (10) and *C*7 motifs form 2D nonbonded molecular assemblies (Figure 3A). As shown in Figure 3B, two of these entries, [(±)-3–2F and (±)-3–3F], also exhibit a high degree of isomorphism (space group *P*2_1_/*c*). These systems, assigned to Form V, assemble components using carboxyl···carboxyl *R*_2_^2^ (8) and N–H···O=C *C*4 hydrogen bond contacts creating 2D motifs. A third phase (Form VI, space group *Cc*, Figure 3D) involving **3**-2F/**3**-3F also forms *R*_2_^2^ (8) and *C*4 motifs, but the quasienantiomeric building blocks create a distinct crystal organization to Form V (Figure 3C).

**Figure 3:**
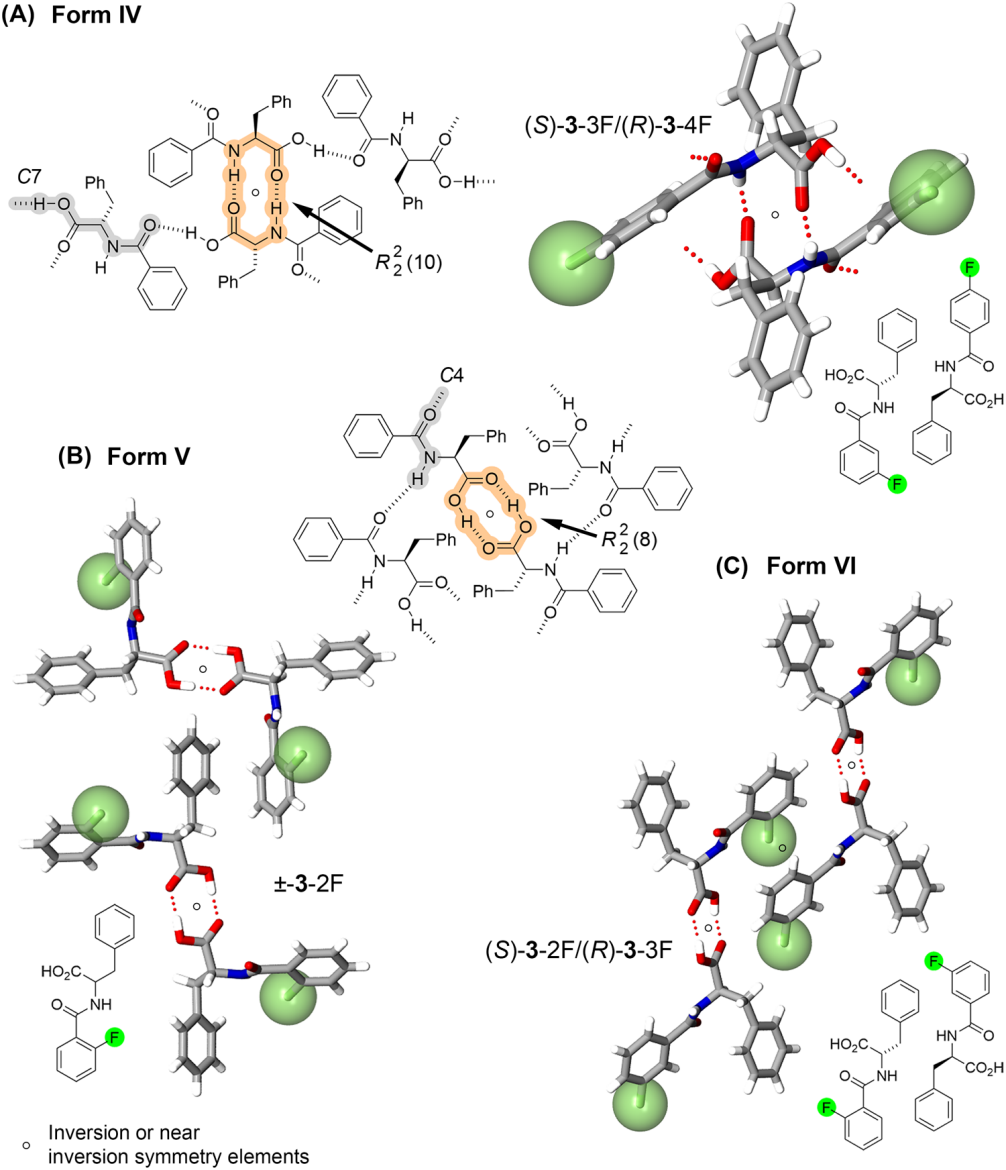
(A) Molecular diagram of Form IV and crystal structure of quasiracemate (*S*)-**3**-3F/(*R*)-**3**-4F showing *R*_2_^2^ (10) and *C*7 hydrogen-bond motifs. (B,C) Forms V and VI utilizing *R*_2_^2^ (10) and *C*4 nonbonded contacts.

Table 3 quantifies the observed structural correlations for the benzoyl phenylalanine **3** systems. The CSD-CPSS assessment of these structures reveals that Forms IV and V correspond to clusters comprising 15 molecules with favorable RMS values, indicating strong correlations between molecular alignment.

**Table 3:**
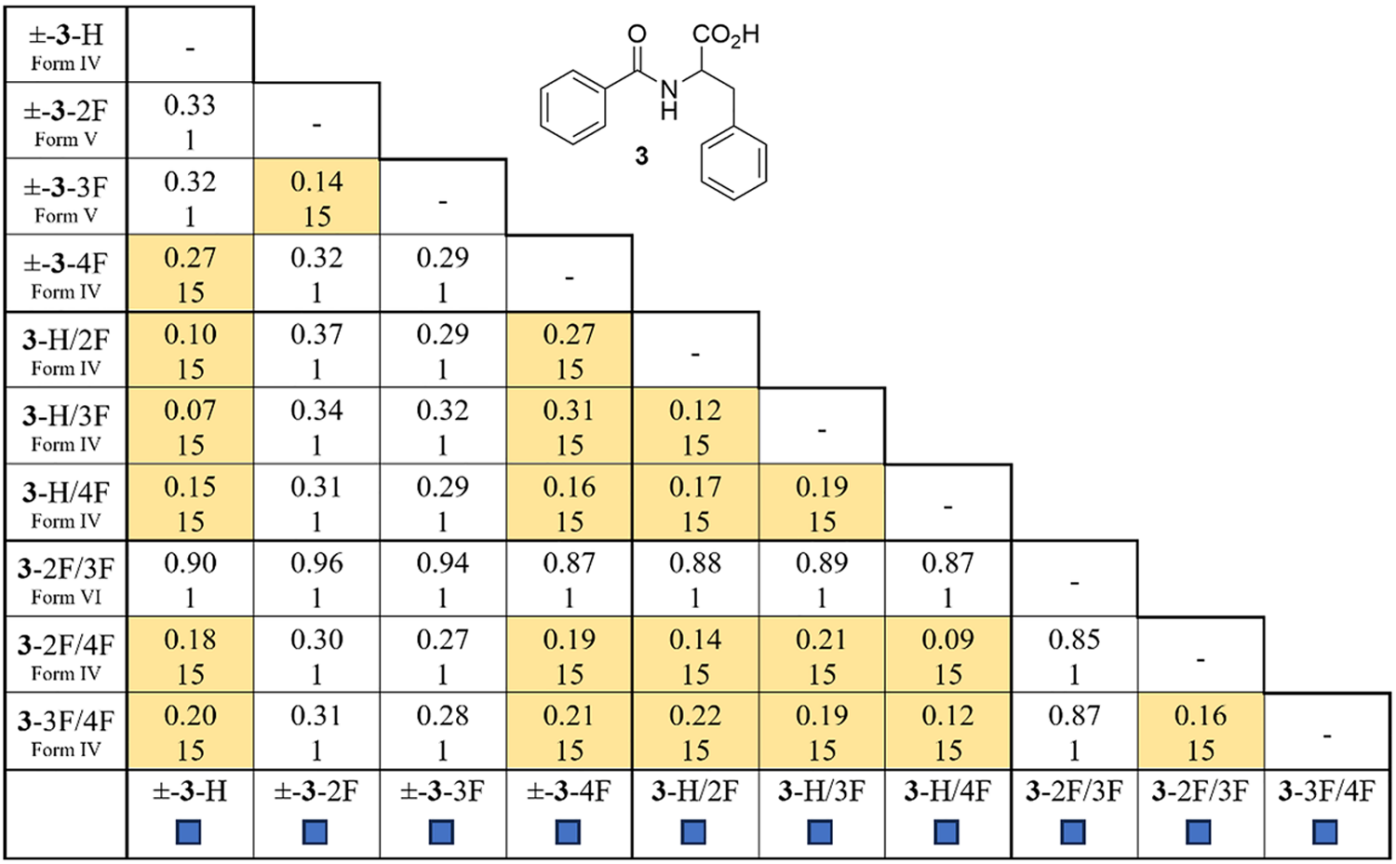
CCDC Mercury Crystal Packing Similarity Search and Hot Stage Results for Benzoyl Phenylalanine 3^a^

a Cell values indicate root-mean-square agreement of the structural pair (top) (maximum 15 molecules). The light and dark-shaded cells indicate entries with 3–9 and 10–15 molecules in the cluster, respectively. Outcomes from hot stage experiments are represented by dark (racemic and quasiracemic formation) and light-colored (conglomerate formation) boxes.

### Crystal Lattice Energy Calculations

The data reported in Tables 1–3 offer direct measures of the structural similarities observed in the crystal structures of **1** – **3**. These structural relationships broadly translate to comparable trends in the computationally derived crystal lattice energies (*E*_Latt_) using Crystal Explorer^37^ equipped with Gaussian16 ^38^ (Table 4). For diarylamides **1**, the *E*_Latt_ values range from −118 to −152.4 kJ/mol, averaging 139.7 kJ/mol. While this system aligns the components in three distinct crystal patterns (Forms I–III), the *E*_Latt_ values for these nine systems are reasonably comparable. Furthermore, these lattice energy relationships for the racemates and quasiracemates of **1** provide valuable evidence of the structural patterns observed in quasiracemates, which mimic the thermodynamically preferred centrosymmetric alignment found in racemates.

**Table 4:** Crystal Lattice Enthalpies (*E*_Latt_) Derived from Crystal Structures of **1** – **3** Using Crystal Explorer (B3LYP/6-31G­(d,p), Gaussian16)

	diarylamide **1**	naphthylamide **2**	benzoyl phenylalanine **3**
**(±)**-H	–145.8 ^27^	–92.2 ^28^	–180.3
**(±)**-2F	–143.9	–114.8	–180.5
**(±)**-3F	–152.4 ^27^	–121.2	–174.8
**(±)**-4F	–142.3 ^27^	–171.1 ^28^	–188.2
H/2F	–122.8 ^26^		–180.5
H/3F	–145.4 ^27^		–178.2
H/4F	–138.8 ^27^	–157.0 ^28^	–184.1
2*F*/3F			–191.0
2*F*/4F	–118.0		–179.0
3*F*/4F	–147.8		–174.8
*E*_Latt_(ave)	–139.7	–131.3	–180.6

The *E*_Latt_ values for the naphthylamide **2** system represent diverse *E*_Latt_ landscapes with the most significant difference (Δ*E*_Latt_ = 64.8 kJ/mol) associated with (±)-**2**-H (*E*_Latt_ = −92.2 kJ/mol) and **2**-H/**2**-4F (*E*_Latt_ = −157.0 kJ/mol). It is interesting to note that the lowest *E*_Latt_ values correlate with the highly modulated (±)-**2**-H and disordered (±)-**2**-2F and (±)-**2**-3F structures, and the structures ((±)-2–4F and 2-H/2–4F) with increased lattice energies lack these crystallographic anomalies. It was previously noted with several of these naphthylamide structures and other derivatives that crystals consisting of **2** improve crystal lattice stabilization compared to **1** due to the increased size of the molecular framework.^28^

By contrast, the crystal structures of **3** consistently demonstrated high *E*_Latt_ and *E*_Latt_(ave) values. The combination of CCDC–CCSP and *E*_Latt_ data provides critical insight into the role of hydrogen bonds in creating predictable crystal landscapes that promote isomorphism and quasiracemate formation.

### Hot Stage Thermomicroscopy

Efforts from our group^26,27,29^ and others^47^ have shown the utility of the hot stage thermomicroscopy technique for uncovering the details of crystallization events. This approach, described by Kofler^48^ in the 1950s, involves the contact fusion method, where samples are delivered to a glass slide and coverslip via the melt. When heated, the interface between materials develops a thermal signature consistent with the molecular recognition events unique to each system.

Figure 4 provides several examples of generated video-assisted hot stage micrographs, which display three snapshots show melting and crystallization events during the heating process. For (*S*)-**1**-3F/(*R*)-**1**-4F, the micrographs indicate that the initial interface between the solid components, upon heating, developed into a eutectic region of melted materials, signifying the formation of a conglomerate[Fn fn1] and unsuccessful cocrystallization of the components (Figure 4A). Similar thermal processing of the (*S*)-**2**-4F and (*R*)-**2**-4F enantiomers and (*S*)-**2**-H and (*R*)-**2**-4F quasienantiomers systems gave different thermal signatures (Figure 4B,C), where the new crystalline phases, accompanied by two eutectic regions, indicate the formation racemic or quasiracemic materials. Several important advantages of this diagnostic tool include the use of small material quantities. Thermal cycling of successful systems is frequently accompanied by further growth of the racemic or quasiracemic region, providing additional support for their formation. Additionally, some materials initially deposited on the glass slide-coverslip setup do not readily recrystallize from the melt. In these cases, we have observed that recrystallization can be induced by applying pressure, varying the temperature, or adding seed crystals to the side of the coverslip. Figure 4D shows such a system, (*R*)-**3**-H/(*S*)-**2**–3F, where the (*R*)-**3**-H component did not recrystallize. Nevertheless, the system produced a distinct quasiracemic crystalline phase. Such observations are important because they draw attention to the utility and potential of the hot stage method as a valuable technique for examining the crystal growth process of multicomponent systems.

**Figure 4:**
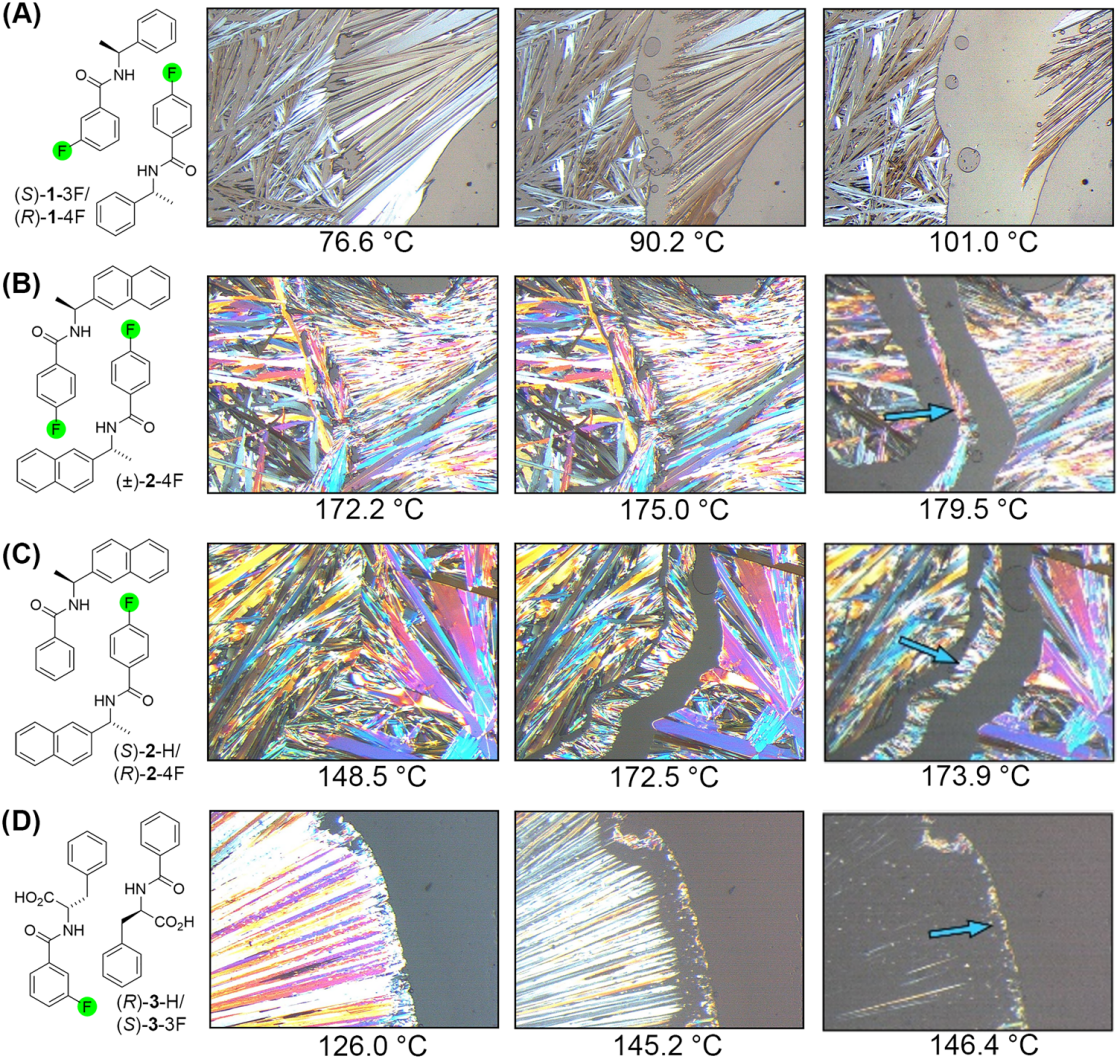
Hot stage thermomicroscopy micrographs of (A) (*S*)-**1**-3F/(*R*)-**1**-4F, (A), (B) (±)-**2**-4F, (C) (*S*)-**2**-H/(*R*)-**2**-4F, (D) (*R*)-**3**-H/(*S*)-**3**-3F. Arrows indicate racemic or quasiracemic formation.

The data presented in Tables 1–3 outline the outcomes from processing all racemic and quasiracemic component combinations for the **1** – **3** molecular frameworks using the hot stage method (30 total). These results demonstrate that crystals grown from the melt were more successful than our efforts with solution growth for X-ray crystallographic assessment. For benzoyl phenylalanine **3**, processing each of the ten racemic and quasiracemic systems achieved samples of sufficient quality for crystallographic assessment. This observation is consistent with the ten crystal structures acquired for these molecular targets. Fewer cocrystallization events were observed with the **1** and **2** frameworks. For diarylamide **1**, four entries – all quasiracemic materials (**1**-H/**1**-4F, **1**-2F/**1**-3F, **1**-2F/**1**-4F, **1**-3F/**1**-4F) – lacked cocrystallization events, while naphthylamide **2** did not show success with the **2**-H/**2**-2F and **2**-H/**2**-3F quasiracemates.

### Combined Analysis

Results from the thermal, crystallographic, and computational investigations of the three targeted systems raise important questions about the molecular recognition processes. Why do some systems readily form quasiracemates via solution and melt cocrystallization, while other materials, when combined, are less effective at creating bimolecular assemblies? One possible answer lies in the stability of the crystal lattice. In the case of benzoyl phenylalanine **3**, the increased ability of the molecular framework to form nonbonded contacts has a profound effect on quasiracemate formation, where all possible racemates and quasiracemates (H/F and F/F) form. Systems **1** and **2**, to a lesser extent, produce quasiracemic compounds from the melt. The hot stage micrographs for framework **2** indicate greater quasiracemate success compared to those of framework **1**, where conglomerates occur in two of the systems for framework **2** (**2**-H/2F and **2**-H/3F) and in four systems for framework **1** (**1**-H/4F, **1**-2F/3F, **1**-2F/4F, and **1**-3F/4F). These results follow a similar trend to the calculated crystal lattice enthalpies (Table 4), with benzoyl phenylalanine **3** being more stable than naphthylamide **2**, and **2** capable of more enthalpically favorable crystal forms than those of arylamide **1**.

Because solvent- and melt-based crystal growth studies can differ significantly in their nucleation and growth processes, the variety of outcomes from these methods, when applied to **1** – **3**, is somewhat anticipated. Each of the four racemates and six quasiracemates of **3** resulted in crystal structures (10 total), with nine structures for diarylamide **1**, and only five structures for naphthylamide **2**. While the data provided in Tables 1–4 may suggest an energetic preference for the crystal forms of naphthylamide **2** over diarylamide **1**, solution growth using **2** proved quite challenging in this work and a previous study.^28^ The success rate of quasiracemic crystal growth for **2** was low despite multiple attempts using various growth techniques and solvent systems.

Another related question is whether the molecular recognition process involving the quasiracemates of **1** – **3** favors the assembly of specific pairs of chiral fluoro regioisomers. At the outset of this study, such a structural bias seemed possible, if not likely, since each 2-, 3-, and 4-fluoro homochiral derivative exhibits distinct spatial and electronic properties. Nevertheless, the patterns within the hot stage and crystallographic data for the three systems lack a clear preference for pairing these quasiracemic components.

### Structural Boundaries – Chloro Regioisomeric Quasiracemates

This study examined all possible quasienantiomeric F/F and H/F pairs using frameworks **1** – **3**. All F/F positional isomers for benzoyl phenylalanine **3** achieved quasiracemate formation, and the analogous systems for **1** and **2** to a lesser extent. While this trend closely follows the observed thermodynamic stability of the crystal structures, the structural limit to pairing regioisomeric quasienantiomers with these systems remains unclear. While the percent difference in H and F volumes (%Δ*V*) is 59.5%, their contributions to molecular volume are relatively small. For example, in the case of the **3**-H and **3**-4F molecules in quasiracemate **3**-H/**3**-4F, the analogous H and F groups contribute 2.4 and 5.6%, respectively, as determined using the MolInfo command provided in OLEX2.^33^

To further probe the structural margin of quasiracemate formation using quasienantiomeric positional isomers, this study also examined the effects of cocrystallizing several chlorine derivatives of **3**. When considering the H and F substituents, chlorine is the next largest monatomic monovalent functional group, with an atomic volume of 22.5 Å^3^ (%Δ*V* for H and Cl = 103.0%). This increase in volume was then implemented in the study by assessing the racemates and positional quasiracemates of three chloro-substituted isomers (*i.e*., **3**-2F/**3**-3F, **3**-2F/**3**-4F, **3**-3F/**3**-4F). Crystal growth from the melt was attempted for each of these systems. Each examined racemate – (±)-**3**-2Cl,(±)-**3**-3Cl, (±)-**3**-4Cl – produced crystal structures and hot stage micrographs indicating successful cocrystal formation (see Supporting Information, Section 2). Conversely, hot stage processing using regioisomeric quasienantiomers gave conglomerates with no indication that the targeted quasiracemates formed.

## Conclusions

This systematic study has shown that constructing quasiracemic materials by pairing positional isomeric quasienantiomers is possible. By examining quasiracemates composed of components that differ in their molecular framework and fluorine group placement, the role of molecular topology and crystal lattice stabilization (*E*_Latt_) in cocrystallization can be assessed. The baseline crystal structures of the racemates and H/F quasiracemates were also studied. Many of the crystal structures for **1** – **3** exhibited isomorphism, as evident from their similar unit cell parameters, crystal packing, and crystallographic data sets. Benzoyl phenylalanine **3**, designed with additional hydrogen bond donor and acceptor sites than diarylamide **1** and naphthylamide **2**, showed a high quasiracemate formation hit rate using melt and solution crystal growth techniques. This observation is also consistent with the higher computational *E*_Latt_ values for **3**. The quasiracemates of **3** derived from Cl regioisomeric quasienantiomers were unsuccessful, indicating an upper structural limit to quasiracemate formation. Results from this study emphasize the importance of quasiracemate component selection, where molecular framework and the details of the imposed structural difference often contribute in detailed ways to the recognition profiles of these cocrystalline materials.

## Supplementary Material



## Footnotes

‡ Racemic materials consist of equimolar amounts of enantiomers. Quasiracemates, structurally similar to true racemates, consist of pairs of chemically unique, near-mirror symmetric compounds with opposing chirality. Conglomerate formation occurs when the crystallization of racemic or quasiracemic mixtures results in the spontaneous generation of individual enantioenriched crystals. (Jaques, J.; Collet, A.; Wilen, S. H. *Enantiomers*, *Racemates*, *and Resolutions*; Wiley: New York, 1981)

## Notes

Additional experimental details, including synthetic details, NMR data, hot stage micrographs, crystallographic data, and hydrogen-bond parameters (PDF)

## Abbreviations

CCDC: Cambridge Crystallographic Data Centre
CE: Crystal Explorer
CPSS: Crystal Packing Similarity Search
CSD: Cambridge Structural Database
