# Supplementary material for: Regioisomeric Quasiracemates – Fluorinated Diarylamide, Naphthylamide, and Benzoyl Phenylalanine Systems
Source: Cryst Growth Des. 2025 Jul 16;25(15):6098–108. doi: 10.1021/acs.cgd.5c00550 (PMC12333013; doi:10.1021/acs.cgd.5c00550)
Supplement: Supplementary file 1 [file cg5c00550_si_001.pdf]

Supporting Information for: Regioisomeric Quasiracemates – Fluorinated Diarylamide, Naphthylamide, and Benzoyl Phenylalanine Systems.

Mu Mu W. Dun, Michaela N. Gao, Ethan C. Vyhmeister, and Kraig A. Wheeler
Department of Chemistry, Whitworth University, 300 West Hawthorne Road, Spokane, Washington, 99251, USA.

Supplementary Information

**Table of Contents**	**Page**
S1. Experimental Details	2
S2. Hot Stage Thermomicroscopy	6
S3. Single-Crystal X-ray Crystallography	13
References	22

S1. Experimental Details

General Considerations: The synthesis of naphthylamide (**2**)[1] and benzoyl phenylalanine (**3**)[2] followed previously described synthetic protocols for related compounds. All chemicals and solvents were purchased from MilliporeSigma or VWR Scientific and used as received without further purification unless stated otherwise. ^{1}H NMR spectral data were recorded with a 400 MHz JEOL 400SS spectrometer using the Delta software (4.3.6.0). Spectral data are referenced using the solvent residual signal as an internal standard, and chemical shift values are expressed as δ values (ppm) and the value of coupling constants (*J*) in Hertz (Hz). The following abbreviations were used for signal multiplicities: *s*, singlet; *d*, doublet; *dd*, doublet of doublets; *t*, triplet; *q*, quartet; *m*, multiplet; and *br*, broad.

Several samples used for cocrystallization and hot stage investigations were retrieved from previous studies conducted in our group. The experimental details and spectroscopic data for these samples are provided in the reference citations listed below.

(*S*)-N-(Benzoyl)methylbenzylamine - (*S*)-1-H[3]

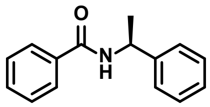

***(R*)-N-(Benzoyl)methylbenzylamine - (*R*)-1-H**[3]

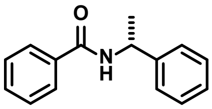

(*S*)-N- (2-Fluorobenzoyl)methylbenzylamine - (*S*)-1-2F[4]

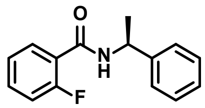

(*R*)-N- (2-Fluorobenzoyl)methylbenzylamine - (*R*)-1-2F[4]

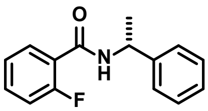

(*S*)-N- (3-Fluorobenzoyl)methylbenzylamine - (*S*)-1-3F[3]

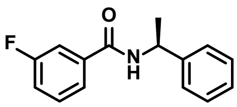

(*R*)-N- (3-Fluorobenzoyl)methylbenzylamine - (*R*)-1-3F[3]

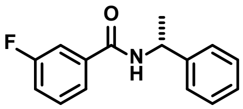

(*S*)-N-(4-Fluorobenzoyl)methylbenzylamine - (*S*)-1-4F[3]

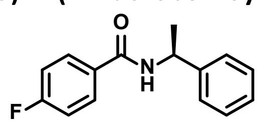

(*R*)-N-(4-Fluorobenzoyl)methylbenzylamine - (*R*)-1-4F[3]

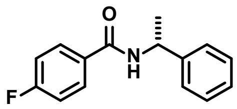

(*S*)-N-(Benzoyl)naphthylethylamine - (*S*)-2-H[1]

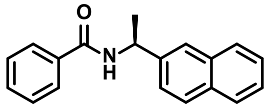

(*R*)-N-(Benzoyl)naphthylethylamine - (*R*)-2-H[1]

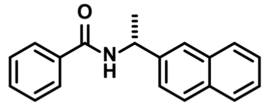

(*S*)-N-(2-Fluorobenzoyl)naphthylethylamine - (*S*)-2-2F

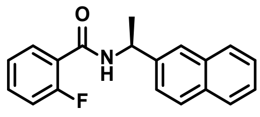

63.1% yield

^{1}H NMR(400 MHz, $CDCl_3$): δ 8.09-7.97 (*m*, 1H, C_{Ar}-H); δ 7.83-7.80 (*m*, 4H, C_{Ar}-H); 7.47-7.43 (*m*, 4H, C_{Ar}-H); 7.16-7.13 (*m*, 2H, C_{Ar}-H); 5.51-5.47 (*m*, 1H, C_{sp3}-H); 1.70 (*d*, *J*=6.8, 3H, CH_3).

(*R*)-N-(2-Fluorobenzoyl)naphthylethylamine - (*R*)-2-2F

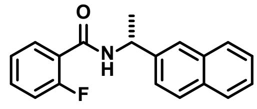

54.8% yield

^{1}H NMR(400 MHz, $CDCl_3$): δ 8.12-7.99 (*m*, 1H, C_{Ar}-H); δ 7.84-7.81 (*m*, 4H, C_{Ar}-H); 7.48-7.43 (*m*, 4H, C_{Ar}-H); 7.17-7.15 (*m*, 2H, C_{Ar}-H); 5.52-5.49 (*m*, 1H, C_{sp3}-H); 1.69 (*d*, *J*=6.8, 3H, CH_3).

(*S*)-N-(3-Fluorobenzoyl)naphthylethylamine - (*S*)-2-3F

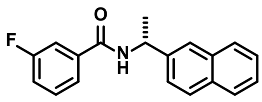

43.3% yield

^{1}H NMR(400 MHz, $CDCl_3$): δ 7.87-7.77 (*m*, 4H, C_{Ar}-H); 7.39-7.35 (*m*, 5H, C_{Ar}-H); 7.30-7.26 (*m*, 1H, C_{Ar}-H); 7.19-7.17 (*m*, 1H, C_{Ar}-H); 5.50-5.48 (*m*, 1H, C_{sp3}-H); 1.70 (*d*, *J*=6.8 Hz, 3H, CH_3).

(*R*)-N-(3-Fluorobenzoyl)naphthylethylamine - (*R*)-2-3F

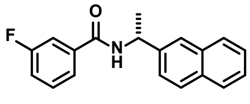

49.7% yield

^{1}H NMR (400 MHz, $CDCl_3$): δ 7.89-7.78 (*m*, 4H, C_{Ar}-H); 7.40-7.35 (*m*, 5H, C_{Ar}-H); 7.30-7.27 (*m*, 1H, C_{Ar}-H); 7.20-7.16 (*m*, 1H, C_{Ar}-H); 5.49-5.47 (*m*, 1H, C_{sp3}-H); 1.70 (*d*, *J*=6.8 Hz, 3H, CH_3).

(*S*)-N-(4-Fluorobenzoyl)naphthylethylamine - (*S*)-2-4F

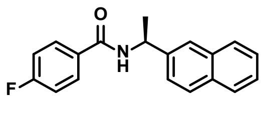

66.8% yield

^{1}H-NMR (400 MHz, $CDCl_3$): δ 7.85-7.77 (*m*, 6H, C_{Ar}-H); 7.51-7.44 (*m*, 3H, C_{Ar}-H); 7.08 (*t*, *J*=8.7 Hz, 2H, C_{Ar}-H); 6.36 *(d, J*=7.3 Hz, 1H, H-N); 5.5 (*m*, C_{sp3}-H); 1.69 (*d*, *J*=6.88 Hz, 3H, CH_3).

(*R*)-N-(4-Fluorobenzoyl)naphthylethylamine - (*R*)-2-4F

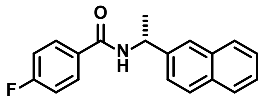

55.7 % yield

^{1}H-NMR (400 MHz, $CDCl_3$): δ 7.84-7.76 (*m*, 6H, C_{Ar}-H); 7.50-7.44 (*m*, 3H, C_{Ar}-H); 7.08 (*t*, *J*=8.7 Hz, 2H, C_{Ar}-H); 6.38 (*d*, *J*=7.32 Hz, 1H, N-H); 5.47 (*q*, *J*=6.88 Hz, 1H, C_{sp3}-H); 1.68 (*d*, *J*=6.9 Hz, 3H, CH_3).

(*R*)-N-(Benzoyl)phenylalanine - (*S*)-3-H[2]

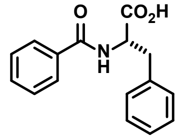

(*S*)-N-(Benzoyl)phenylalanine - (*R*)-3-H[2]

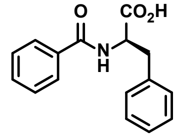

(*S*)-N-(2-Fluorobenzoyl)phenylalanine - (*S*)-3-2F

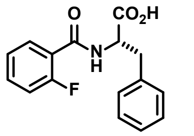

65.5% yield

^{1}H NMR (400 MHz, Acetone-d_6). δ (ppm): 7.85-7.79 (*m*, 1H, C_{Ar}-H); 7.71-7.55 (*m*, 2H, C_{Ar}-H); 7.61-7.21 (*m*, 7H, C_{Ar}-H and N-H); 4.93-4.88 (*m*, 1H, C_{sp3}-H); 3.32 (*dd*, *J* = 5.50, 13.7 Hz, 1H, CH_2); 3.18 (*dd*, *J* = 7.33, 13.7 Hz, 1H, CH_2).

(*R*)-N-(2-Fluorobenzoyl)phenylalanine - (*R*)-3-2F

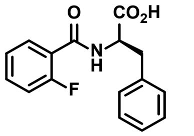

74.0% yield

^{1}H NMR (400 MHz, Acetone-d_6). δ (ppm): 7.86-7.79(*m*, 1H, C_{Ar}-H); 7.56-7.50 (*m*, 2H, C_{Ar}-H); 7.59-7.15 (m, 7H, C_{Ar}-H and N-H); 4.91-4.85 (*m*, 1H, C_{sp3}-H); 3.32 (*dd*, *J* = 5.5, 13.7 Hz, 1H, CH_2); 3.17 (dd, *J* = 7.3, 13.7 Hz, 1H, CH_2).

(*S*)-N-(3-Fluorobenzoyl)phenylalanine - (*S*)-3-3F

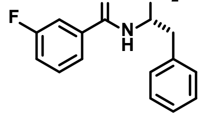

45.3% yield

^{1}H NMR (400 MHz, $CDCl_3$. δ (ppm): 7.41-7.26 (m, 5H, C_{Ar}-H); 7.22-7.18 (m, 3H, C_{Ar}-H); 6.50 (d, *J* = 7.3 Hz, 1H, N-H); 5.04 (*m*, 1H, C_{sp3}-H); 3.30 (dd, *J* = 5.5, 13.7 Hz, 2H, CH_2), 3.17 (dd, *J* = 7.3, 13.7 Hz, 2H, CH_2).

(*R*)-N-(3-Fluorobenzoyl)phenylalanine - (*R*)-3-3F

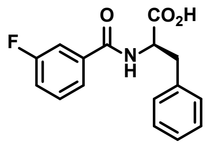

60.0% yield

^{1}H NMR (400 MHz, Acetone-d_6). δ (ppm): 7.83 (*d*, *J* = 7.4 Hz, 2H, N-H); 7.57-7.15 (*m*, 9H, C_{Ar}-H); 4.86-4.77 (*m*, 1H, C_{sp3}-H); 3.34(*dd*, *J* = 5.0, 14.2 Hz, 1H, CH_2); 3.19 (*dd*, *J* = 7.8, 14.2 Hz, 1H, CH_2).

(*S*)-N-(4-Fluorobenzoyl)phenylalanine - (*S*)-3-4F

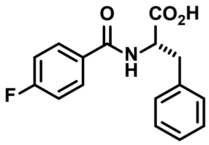

56.9% yield

^{1}H NMR (400 MHz, Acetone-d_6). δ (ppm): 7.88 (*d*, *J* = 5.5 Hz, 2H, C_{Ar}-H); 7.86 (*d*, *J* = 5.5 Hz, 2H, C_{Ar}-H); 7.81 (*d*, *J* = 8.2 Hz, 1H, N-H); 7.34-7.13 (*m*, 5H, C_{Ar}-H); 4.88 (*ddd*, *J* = 5.0, 8.2, 9.6 Hz, 1H, C_{sp3}-H); 3.31 (*dd*, *J* = 5.0, 13.8 Hz, 1H, CH_2), 3.12 (*dd*, *J* = 9.6, 13.8 Hz, 1H, CH_2).

(*R*)-N-(4-Fluorobenzoyl)phenylalanine - (*R*)-3-4F

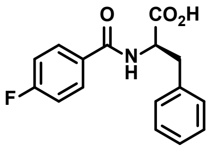

51.5% yield

^{1}H NMR (400 MHz, Acetone-d_6). δ ppm): 7.90 (*d*, *J* = 5.6 Hz, 2H, C_{Ar}-H); 7.86 (*d*, *J* = 5.6 Hz, 2H, C_{Ar}-H); 7.83 (*d*, *J* = 8.4 Hz, 1H, N-H); 7.36-7.11 (m, 5H, C_{Ar}-H); 4.88 (*m*, 1H, C_{sp3}-H); 3.33 (*dd*, *J* = 4.8, 13.8 Hz, 1H, CH_2), 3.13 (*dd*, *J* = 9.2, 13.8 Hz, 1H, CH_2).

S2. Hot-Stage Thermomicroscopy

Hot-Stage Images of Racemic and Quasiracemic Pairs – Diaryalamide (1)

(*S*)-**1**-H<br>(*R*)-**1**-H	Previously reported hot stage data for this system show racemate formation.[3]		
(*S*)-**1**-2F<br>(*R*)-**1**-2F	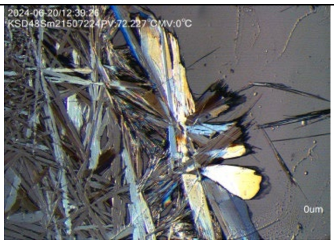<br>72.2°	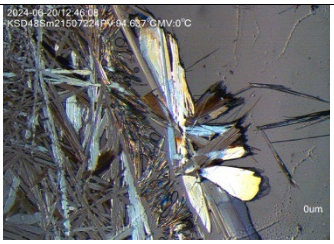<br>94.6°	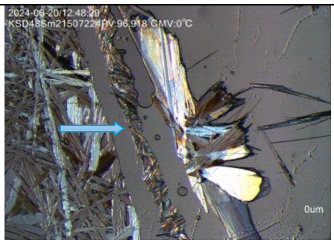<br>96.9°
(*S*)-**1**-3F<br>(*R*)-**1**-3F	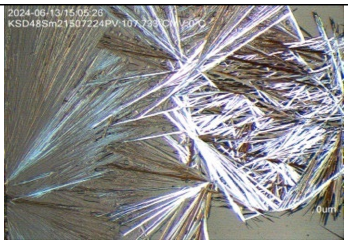<br>107.7°	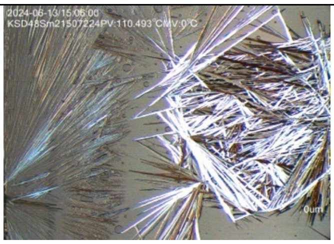<br>110.5°	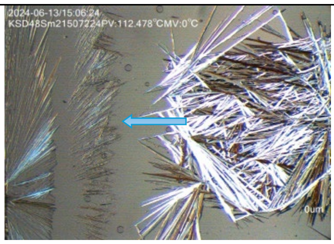<br>112.5°
(*S*)-**1**-4F<br>(*R*)-**1**-4F	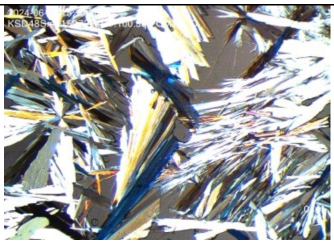<br>100.5°	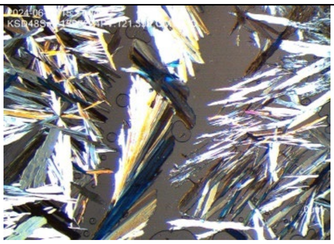<br>121.4°	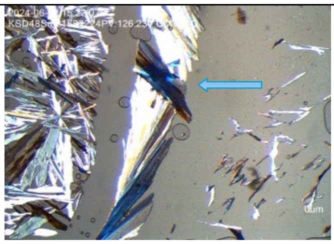<br>126.2°
(*S*)-**1**-H<br>(*R*)-**1**-2F	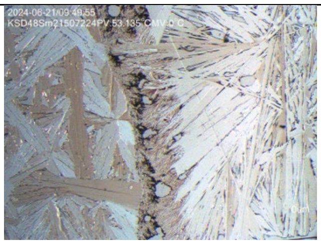<br>53.1°	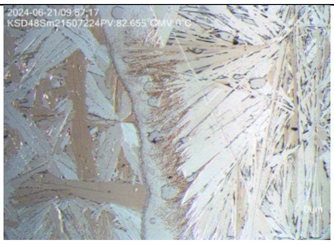<br>82.7°	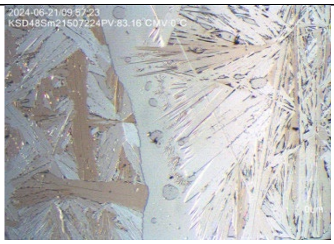<br>83.2°

(*S*)-**1**-H<br>(*R*)-**1**-3F	Previously reported hot stage data for this system show quasiracemate formation.[3]		
(*S*)-**1**-H<br>(*R*)-**1**-4F	Previously reported hot stage data for this system show quasiracemate formation.[3]		
(*S*)-**1**-3F<br>(*R*)-**1**-2F	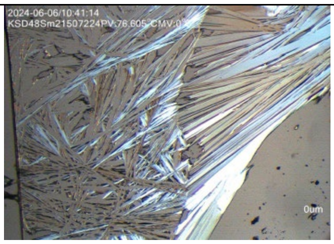<br>76.6°	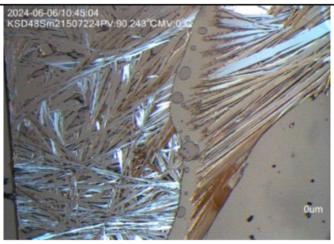<br>90.2°	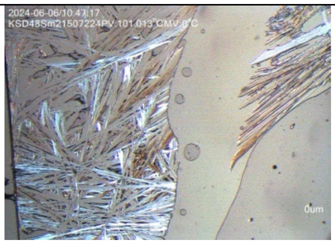<br>101.0°
(*S*)-**1**-4F<br>(*R*)-**1**-2F	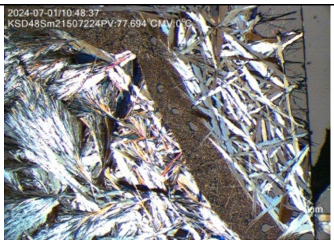<br>77.7°	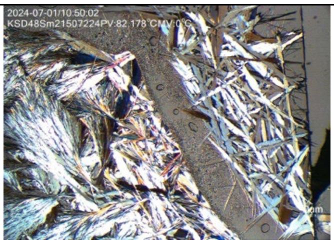<br>82.2°	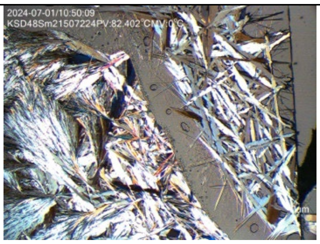<br>82.4°
(*S*)-**1**-4F<br>(*R*)-**1**-3F	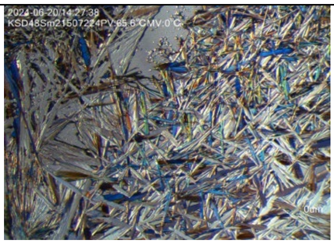<br>65.6°	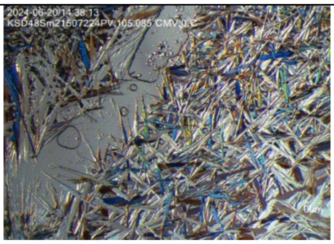<br>105.1°	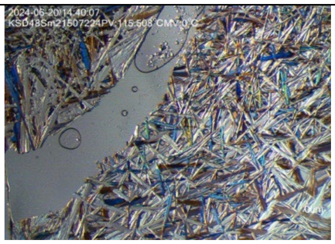<br>115.5°

Hot-Stage Images of Racemic and Quasiracemic Pairs – Naphthylamide (2)

(*S*)-**2**-H<br>(*R*)-**2**-H	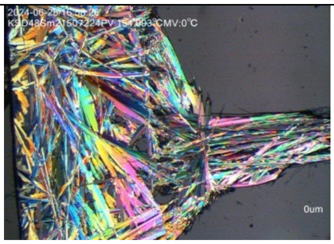<br>154.0°	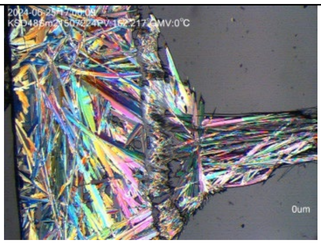<br>162.2°	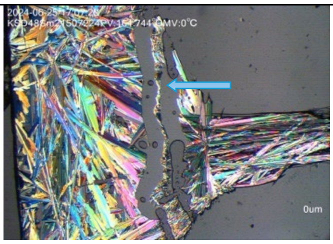<br>164.7°
(*S*)-**2**-2F<br>(*R*)-**2**-2F	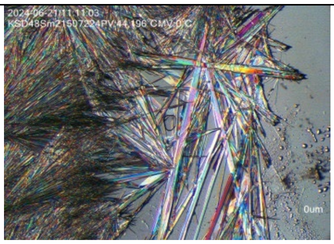<br>44.2°	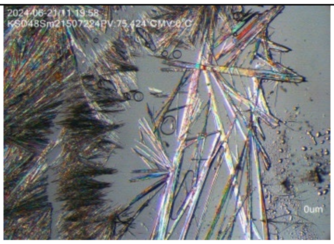<br>75.4°	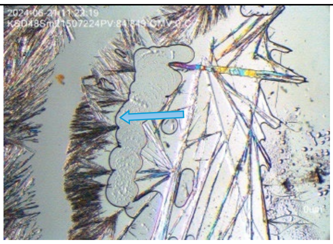<br>84.5°
(*S*)-**2**-3F<br>(*R*)-**2**-3F	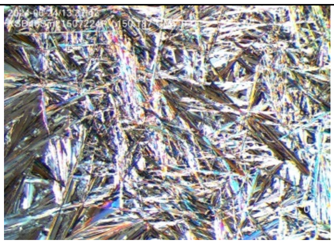<br>150.2°	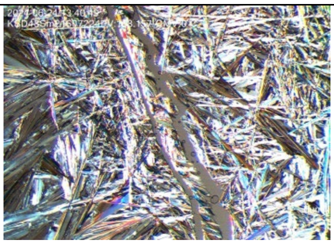<br>158.2°	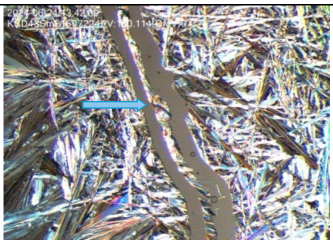<br>160.1°
(*S*)-**2**-4F<br>(*R*)-**2**-4F	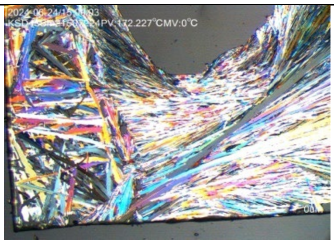<br>172.2°	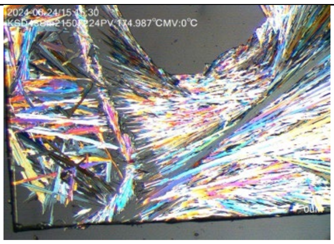<br>175.0°	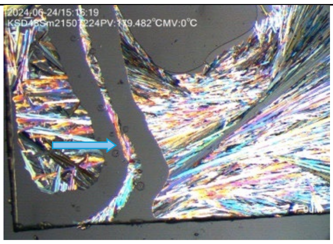<br>179.5°
(*S*)-**2**-2F<br>(*R*)-**2**-H	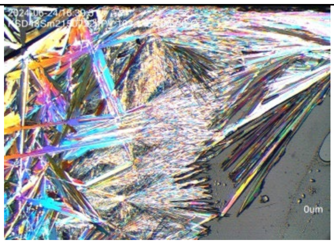<br>103.4°	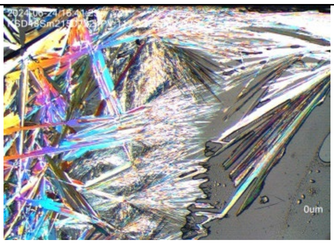<br>117.8°	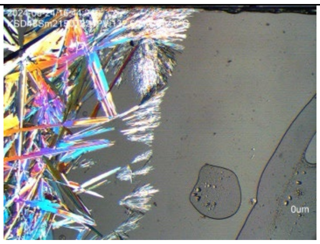<br>132.6°

(*S*)-**2**-H<br>(*R*)-**2**-3F	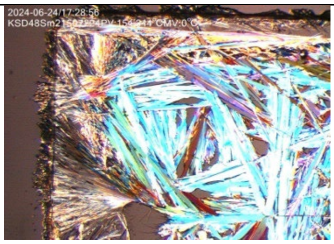<br>154.3°	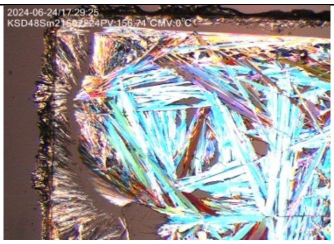<br>156.7°	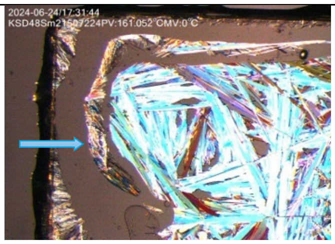<br>161.1°
(*R*)-**2**-H<br>(*S*)-**2**-4F	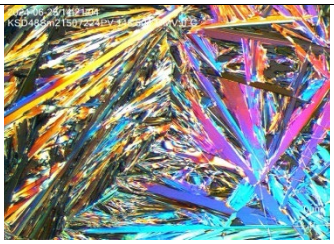<br>148.5°	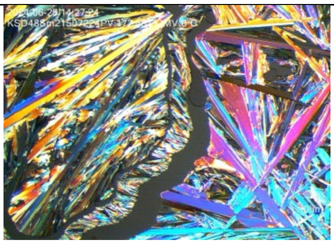<br>172.5°	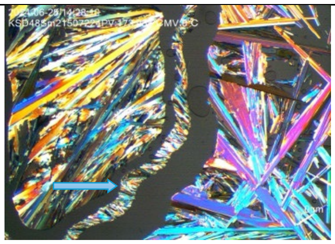<br>173.9°
(*R*)-**2**-2F<br>(*S*)-**2**-3F	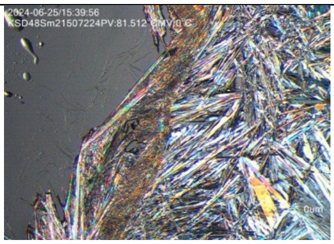<br>81.5°	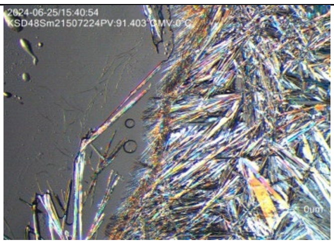<br>91.4°	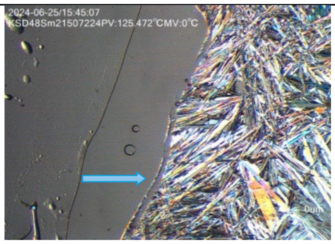<br>125.5°
(*R*)-**2**-2F<br>(*S*)-**2**-4F	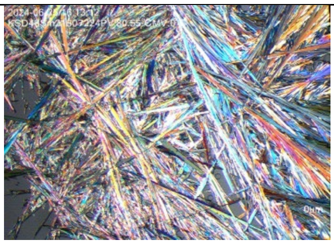<br>80.6°	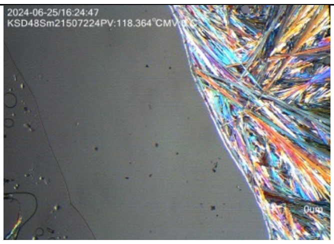<br>118.4°	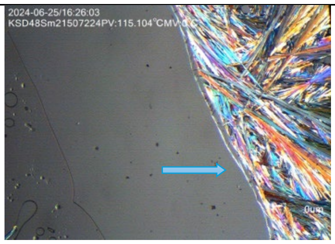<br>115.1°
(*R*)-**2**-3F<br>(*S*)-**2**-4F	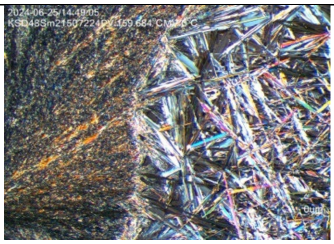<br>159.7°	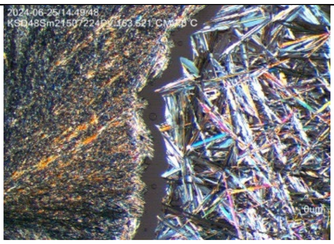<br>163.5°	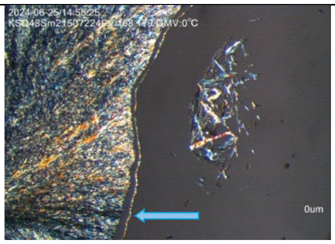<br>168.2°

Hot-Stage Images of Racemic and Quasiracemic Pairs – Phenylalanine (3)

(*S*)-**3**-H<br>(*R*)-**3**-H	Previously reported hot stage data for this system show racemate formation.[2]		
(*S*)-**3**-2F<br>*R*)-**3**-2F	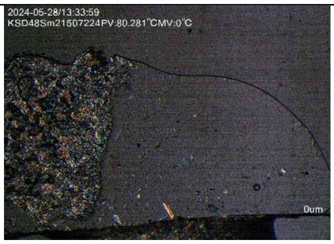<br>83.9°	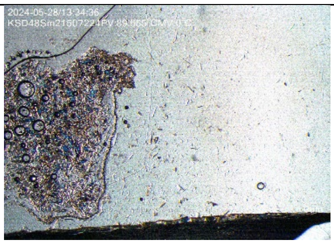<br>89.3°	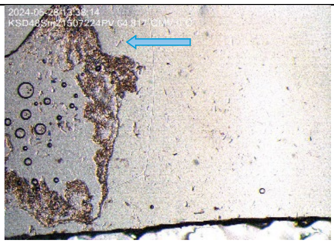<br>105.3°
(*S*)-**3**-3F<br>(*R*)-**3**-3F	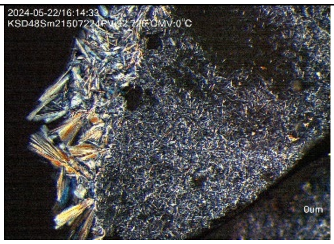<br>92.7°	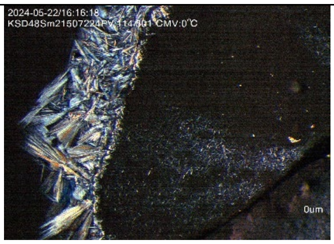<br>159.1°	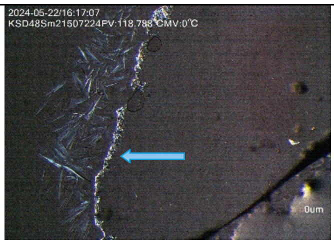<br>163.3°
(*S*)-**3**-4F<br>(*R*)-**3**-4F	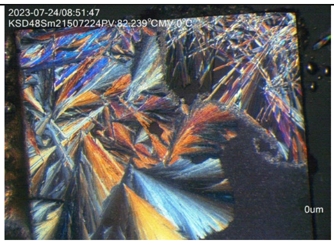<br>82.2°	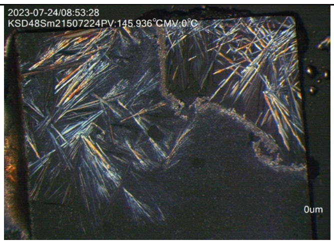<br>145.9°	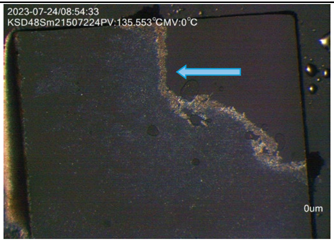<br>153.6°
(*R*)-**3**-H<br>(*S*)-**3**-2F	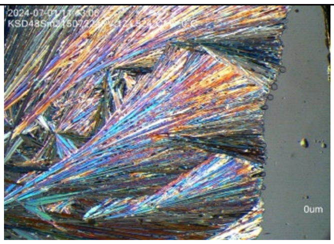<br>124.5°	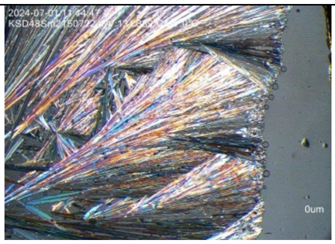<br>134.8°	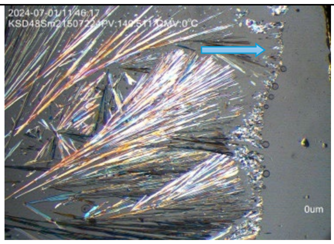<br>140.5°
(*R*)-**3**-H<br>(*S*)-**3**-3F	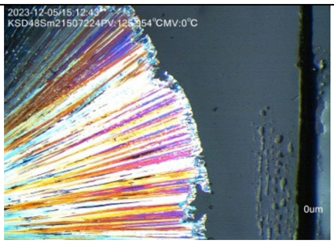<br>126.0°	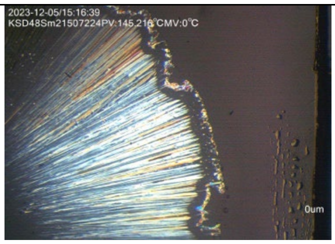<br>130.1°	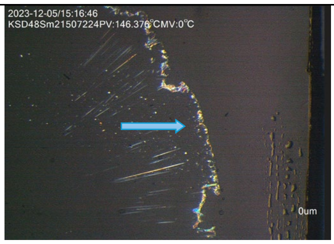<br>135.2°

(*R*)-**3**-H<br>(*S*)-**3**-4F	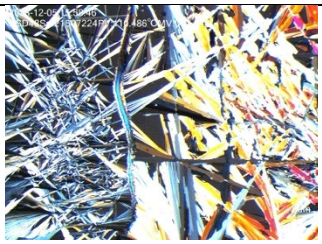<br>110.8°	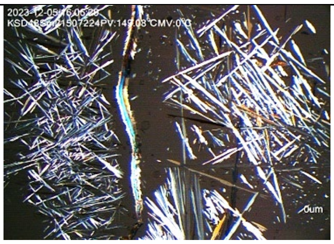<br>149.1°	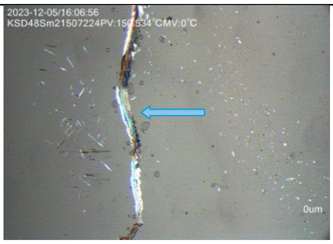<br>150.5°
(*R*)-**3**-2F<br>(*S*)-**3**-3F	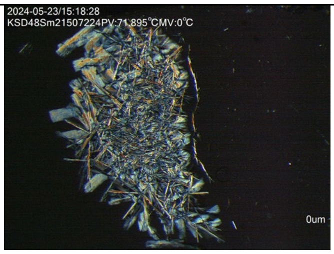<br>80.5°	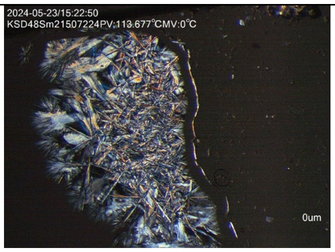<br>84.3°	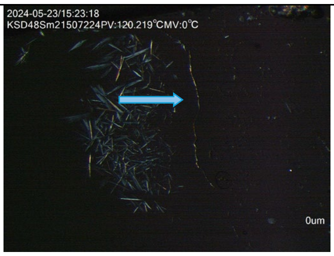<br>85.5°
(*R*)-**3**-2F<br>(*S*)-**3**-4F	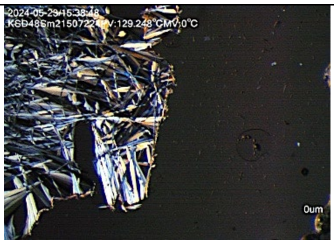<br>129.2°	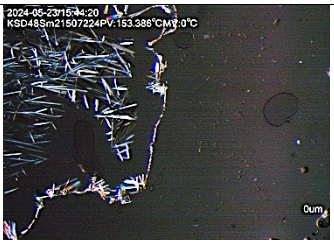<br>153.4°	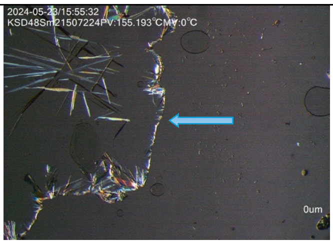<br>155.2°
(*R*)-**3**-3F<br>(*S*)-**3**-4F	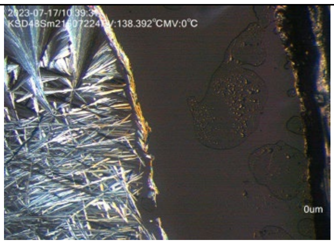<br>138.4°	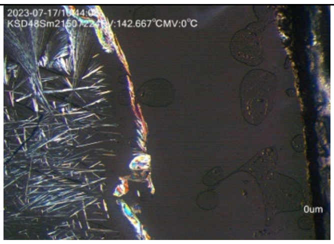<br>142.7°	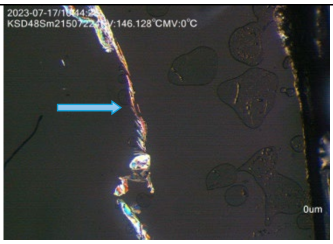<br>146.1°

(*S*)-**3**-2Cl<br>(*R*)-**3**-2Cl	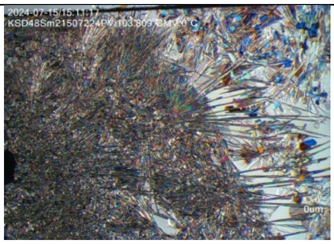<br>103.8°	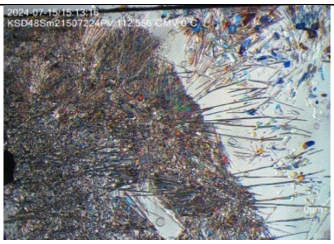<br>112.6°	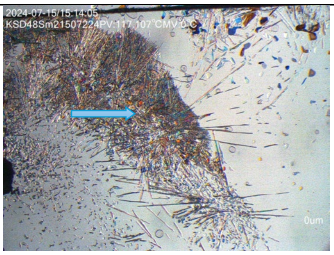<br>117.1°

(*S*)-**3**-3Cl<br>(*R*)-**3**-3Cl	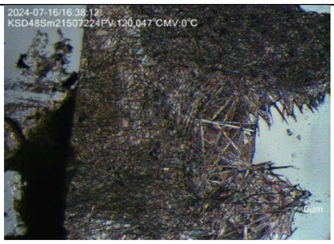<br>120.0°	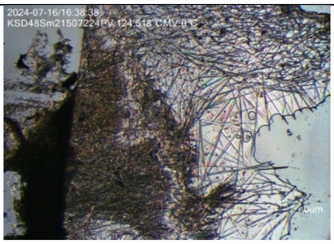<br>124.5°	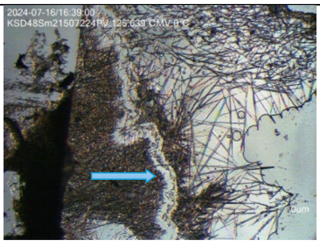<br>125.6°
(*S*)-**3**-4Cl<br>(*R*)-**3**-4Cl	Previously reported hot stage data for this system show racemate formation.[2]		
(*R*)-**3**-2Cl<br>(*S*)-**3**-3Cl	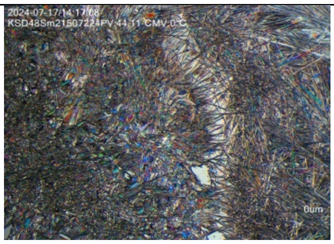<br>44.1°	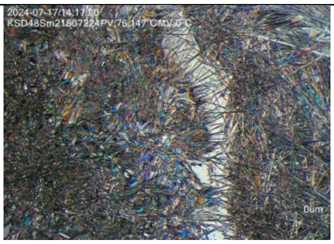<br>76.1°	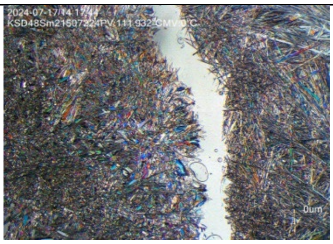<br>111.9°
(*R*)-**3**-2Cl<br>(*S*)-**3**-4Cl	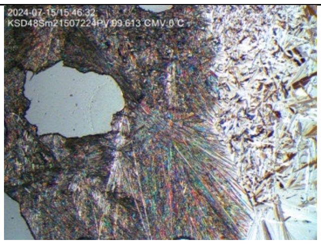<br>99.6°	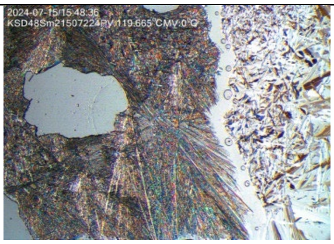<br>119.7°	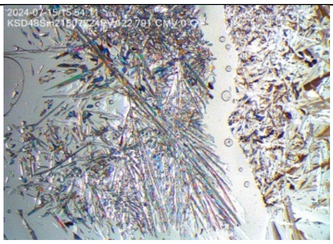<br>122.8°
(*R*)-**3**-3Cl<br>(*S*)-**3**-4Cl	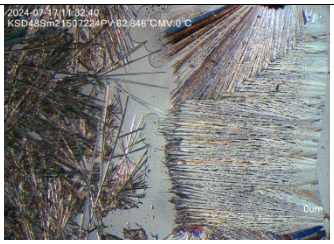<br>62.8°	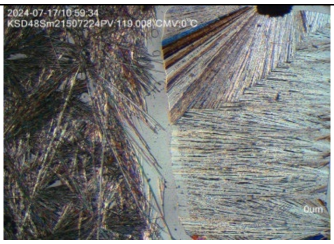<br>119.0°	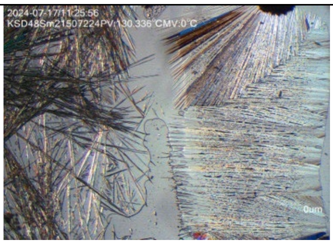<br>130.3°

S3. Single Crystal X-ray Crystallography

Table S1. Crystallographic Data for the Diarylamide (**1**), Naphthylamide (**2**), and Benzoyl Phenylalanine (**3**) Systems.

	(±)-**1**-H[3]	(±)-**1**-2F	(±)-**1**-3F[3]	(±)-**1**-4F[3]
Crystal data				
CCDC deposit no. or refcode	YAYHAL	2442421	YAYGUE	YAYHUF
Empirical formula	$C_{15}H_{15}NO$	$C_{15}H_{14}FNO$	$C_{15}H_{14}FNO$	$C_{15}H_{15}FNO$
Crystal System, space group	Triclinic $P\overline{1}$ (no. 2)	Monoclinic $P2_1/c$ (no. 14)	Monoclinic $P2_1/c$ (no. 14)	Monoclinic $P2_1/c$ (no. 14)
M_r		486.54		
a, Å	5.326	16.3469(6)	23.880	11.491
b, Å	15.374	5.4050(2)	5.212	11.738
c, Å	15.993	27.5555(9)	19.650	9.637
α, deg	64.79	90	90	90
β, deg	85.94	100.236(2)	90.42	106.17
γ, deg	81.51	90	90	90
V, (Å^3)	1171.691	2395.92(15)	2445.618	1248.488
Z, Z'	4, 2	8, 2	8, 2	4, 1
D_{calc} (g cm^{-3})	1.277	1.349	1.321	1.294
μ (mm^{-1}), rad. type		0.779, Cu $K\alpha$		
F_{000}		1024		
temp (K)		100(2)		
Crystal form, color		needle, colorless		
Crystal size, mm		0.38 x 0.20 x 0.08		
Data collection				
Diffractometer		Bruker D8 Venture		
T_{min}/T_{max}		0.652/0.753		
No. of refls. (meas., uniq., and obs.)		44710/4411/3828		
R_{int}		0.0448		
θ_{max} (°)		68.525		
Refinement				
R/R^2_ω (obs data)		0.0382/0.0913		
R/R^2_ω (all data)		0.0469/0.0969		
S		1.061		
No. of refls.		4411		
No. of parameters		343		
$\Delta\rho_{max/min}$ (e·Å^{-3})		0.333/-0.211		
Flack		-		

Table S1. Crystallographic Data for the Diarylamide (**1**), Naphthylamide (**2**), and Benzoyl Phenylalanine (**3**) Systems. (Continued)

	(*S*)-**1**-H/(*R*)-**1**-2F[4]	(*R*)-**1**-H/(*S*)-**1**-3F[3]	(*R*)-**1**-H/(*S*)-**1**-4F[3]
Crystal data			
CCDC refcode	WANLUV	YAYGEO	YAYGAK
Empirical formula	$C_{30}H_{29}FN_2O_2$	$C_{30}H_{29}FN_2O_2$	$C_{30}H_{29}FN_2O_2$
Crystal System, space group	Triclinic $P1$ (no. 1)	Monoclinic $P2_1$ (no. 4)	Monoclinic $P2_1$ (no. 4)
M_r			
a, Å	5.354	19.614	9.663
b, Å	8.382	5.234	11.683
c, Å	13.859	23.553	11.471
α, deg	96.48	90	90
β, deg	93.83	90.41	106.19
γ, deg	105.84	90	90
V, (Å^3)	591.416	2418.180	1243.652
Z, Z'	1, 1	4, 2	2, 1
D_{calc} (g cm^{-3})	1.316	1.287	1.251
μ (mm^{-1}), rad. type			
F_{000}			
temp (K)			
Crystal form, color			
Crystal size, mm			
Data collection			
Diffractometer			
T_{min}/T_{max}			
No. of refls. (meas., uniq., and obs.)			
R_{int}			
θ_{max} (°)			
Refinement			
R/R^2_{ω} (obs data)			
R/R^2_{ω} (all data)			
S			
No. of refls.			
No. of parameters			
$\Delta\rho_{max/min}$ (e·Å^{-3})			
Flack			

Table S1. Crystallographic Data for the Diarylamide (**1**), Naphthylamide (**2**), and Benzoyl Phenylalanine (**3**) Systems. (Continued)

	(*S*)-**1**-2F/(*R*)-**1**-4F	(*S*)-**1**-3F/(*R*)-**1**-4F
Crystal data		
CCDC deposit no.	2442422	2442423
Empirical formula	$C_{30}H_{29}F_2N_2O_2$	$C_{30}H_{28}F_2N_2O_2$
Crystal System, space group	Triclinic *P*1 (no. 1)	Monoclinic $P2_1$ (no. 4)
M_r	487.55	486.54
a, Å	5.3561(3)	9.4979(4)
b, Å	8.3074(4)	5.9365(2)
c, Å	14.0304(7)	21.7074(8)
α, deg	94.410(2)	90
β, deg	93.727(2)	98.575(1)
γ, deg	105.201(2)	90
V, (Å^3)	598.30(5)	1210.27(8)
Z, *Z'*	1, 1	2, 1
D_{calc} (g cm^{-3})	1.353	1.335
μ (mm^{-1}), rad. type	0.780, Cu $K\alpha$	0.094, Mo $K\alpha$
F_{000}	257	512
temp (K)	100(2)	100(2)
Crystal form, color	needle, colorless	plate, colorless
Crystal size, mm	0.25 x 0.16 x 0.05	0.39 x 0.11 x 0.05
Data collection		
Diffractometer	Bruker D8 Venture	Bruker D8 Venture
T_{min}/T_{max}	0.670/0.753	0.652/.753
No. of refls. (meas., uniq., and obs.)	24764/4277/4196	56996/4426/3963
R_{int}	0.0286	0.0558
θ_{max} (°)	68.658	25.343
Refinement		
R/R^2_{ω} (obs data)	0.0338/0.0903	0.0387/0.0883
R/R^2_{ω} (all data)	0.0344/0.0908	0.0460/0.0913
S	1.040	1.095
No. of refls.	4277	4428
No. of parameters	438	333
$\Delta\rho_{max/min}$ (e·Å^{-3})	0.276/-0.236	0.242/-0.218
Flack	0.08(3)	0.2(2)

Table S1. Crystallographic Data for the Diarylamide (**1**), Naphthylamide (**2**), and Benzoyl Phenylalanine (**3**) Systems. (Continued)

	(±)-**2**-H[1]	(±)-**2**-2F	(±)-**2**-3F	(±)-**2**-4F[1]
Crystal data				
CCDC deposit no. or refcode	WABJUI	2442424	2442425	WABFEO
Empirical formula	$C_{19}H_{17}NO$	$C_{19}H_{16}FNO$	$C_{19}H_{16}FNO$	$C_{19}H_{16}FNO$
Crystal System, space group	Triclinic $P\bar{1}$ (no. 2)	Monoclinic *Cc* (no. 9)	Triclinic *C*2/*c* (no. 15)	Triclinic $P\bar{1}$ (no. 2)
M_r	275.34	293.33	293.33	293.33
a, Å	10.22	5.0421(2)	19.5076(7)	9.930
b, Å	30.04	19.2701(9)	5.0838(2)	10.224
c, Å	57.34	30.1744(14)	29.4111(11)	15.391
α, deg	89.58	90	90	74.36
β, deg	87.45	94.709(3)	90.483(2)	86.70
γ, deg	80.28	90	90	72.91
V, (Å^3)	17326.16	2921.9(2)	2116.68(19)	1437.81
Z, *Z'*	48, 24	8, 2	8, 1	4, 2
D_{calc} (g cm^{-3})	1.267	1.334	1.336	1.355
μ (mm^{-1}), rad. type		0.737, Cu $K\alpha$	0.739, Cu $K\alpha$	
F_{000}		1232	1232	
temp (K)		100(2)	100(2)	
Crystal form, color		needle, colorless	needle, colorless	
Crystal size, mm		0.28 x 0.02 x 0.02	0.39 x 0.07 x 0.04	
Data collection				
Diffractometer		Bruker D8 Venture	Bruker D8 Venture	
T_{min}/T_{max}		0.6042/0.7319	0.762/0.975	
No. of refls. (meas., uniq., and obs.)		23744/5167/4543	24016/2668/2388	
R_{int}		0.0598	0.0562	
θ_{max} (°)		68.493	68.285	
Refinement				
R/R^2_{ω} (obs data)		0.0471/0.1012	0.0386/0.0898	
R/R^2_{ω} (all data)		0.0574/0.1090	0.0440/0.0939	
S		1.060	1.093	
No. of refls.		5167	2668	
No. of parameters		587	306	
$\Delta\rho_{max/min}$ (e·Å^{-3})		0.207/-0.214	0.137/-0.213	
Flack		-	-	

Table S1. Crystallographic Data for the Diarylamide (**1**), Naphthylamide (**2**), and Benzoyl Phenylalanine (**3**) Systems. (Continued)

	(*S*)-**2**-H/(*R*)-**2**-4F[1]
Crystal data	
CCDC refcode	WABFAK
Empirical formula	$C_{38}H_{33}FN_2O_2$
Crystal System, space group	Triclinic *P*1 (no. 1)
M_r	568.66
a, Å	9.852
b, Å	10.23
c, Å	15.38
α, deg	76.29
β, deg	88.60
γ, deg	72.36
V, (Å^3)	1432.84
Z, *Z'*	2, 2
D_{calc} (g cm^{-3})	1.318
μ (mm^{-1}), rad. type	
F_{000}	
temp (K)	
Crystal form, color	
Crystal size, mm	
Data collection	
Diffractometer	
T_{min}/T_{max}	
No. of refls. (meas., uniq., and obs.)	
R_{int}	
θ_{max} (°)	
Refinement	
R/R^2_{ω} (obs data)	
R/R^2_{ω} (all data)	
S	
No. of refls.	
No. of parameters	
$\Delta\rho_{max/min}$ (e·Å^{-3})	
Flack	

Table S1. Crystallographic Data for the Diarylamide (**1**), Naphthylamide (**2**), and Benzoyl Phenylalanine (**3**) Systems. (Continued)

	(±)-**3**-H[2]	(±)-**3**-2F	(±)-**3**-3F	(±)-**3**-4F
Crystal data				
CCDC deposit no. or refcode	ECAMIE01	2442426	2442427	2442428
Empirical formula	$C_{16}H_{15}NO_3$	$C_{16}H_{14}FNO_3$	$C_{16}H_{14}FNO_3$	$C_{16}H_{14}FNO_3$
Crystal System, space group	Monoclinic $P2_1/c$ (no. 14)	Monoclinic $P2_1/c$ (no. 14)	Monoclinic $P2_1/c$ (no. 14)	Monoclinic $P2_1/c$ (no. 14)
M_r	269.29	287.28	287.28	287.28
a, Å	11.226	5.2578(2)	5.1896(2)	11.0565(4)
b, Å	9.008	15.0598(5)	15.3337(5)	9.2604(3)
c, Å	14.010	17.5453(6)	17.6004(6)	14.2353(5)
α, deg	90	90	90	90
β, deg	107.10	96.320(2)	96.816(2)	108.470(1)
γ, deg	90	90	90	90
V, (Å^3)	1354.119	1380.82(8)	1390.67(8)	1382.44(8)
Z, Z'	4, 1	4, 1	4, 1	4, 1
D_{calc} (g cm^{-3})	1.321	1.382	1.367	1.380
μ (mm^{-1}), rad. type		0.878, Cu $K\alpha$	0.872, Cu $K\alpha$	0.877, Cu $K\alpha$
F_{000}		600	600	600
temp (K)		100(2)	100(2)	100(2)
Crystal form, color		Needle, colorless	needle, colorless	needle, colorless
Crystal size, mm		0.38 x 0.05 x 0.03	0.18 x 0.09 x 0.05	0.24 x 0.18 x 0.12
Data collection				
Diffractometer		Bruker D8 Venture	Bruker D8 Venture	Bruker D8 Venture
T_{min}/T_{max}		0.6617/0.7531	0.6736/0.7531	0.6997/0.7535
No. of refls. (meas., uniq., and obs.)		24812/2527/2319	26798/2557/2357	41870/2720/2542
R_{int}		0.0413	0.0332	0.0286
θ_{max} (°)		68.279	68.473	72.089
Refinement				
R/R^2_ω (obs data)		0.0446/0.1020	0.0381/0.0874	0.0315/0.0763
R/R^2_ω (all data)		0.0495/0.1055	0.0416/0.0899	0.0340/0.0780
S		1.088	1.084	1.070
No. of refls.		2527	2557	2720
No. of parameters		196	206	196
$\Delta\rho_{max/min}$ (e·Å^{-3})		0.480/-0.230	0.295/-0.217	0.292/-0.227
Flack		-	-	-

Table S1. Crystallographic Data for the Diarylamide (**1**), Naphthylamide (**2**), and Benzoyl Phenylalanine (**3**) Systems. (Continued)

	(*S*)-**3**-H/(*R*)-**3**-2F	(*R*)-**3**-H/(*S*)-**3**-3F	(*S*)-**3**-H/(*R*)-**3**-4F
Crystal data			
CCDC deposit no.	2442429	2442430	2442431
Empirical formula	$C_{32}H_{29}FN_2O_6$	$C_{32}H_{29}FN_2O_6$	$C_{32}H_{29}FN_2O_6$
Crystal System, space group	Monoclinic $P2_1$ (no. 4)	Monoclinic $P2_1$ (no. 4)	Monoclinic $P2_1$ (no. 4)
M_r	556.57	556.57	556.57
a, Å	11.2079(9)	11.2924(2)	11.1941(5)
b, Å	9.1005(7)	9.0029(2)	9.1213(4)
c, Å	13.9771(11)	14.1050(3)	14.1615(6)
α, deg	90	90	90
β, deg	106.714(3)	106.765(1)	108.026(2)
γ, deg	90	90	90
V, (Å^3)	1365.40(19)	1373.03(5)	1374.98(10)
Z, *Z'*	2, 1	2, 1	2, 1
D_{calc} (g cm^{-3})	1.354	1.346	1.344
μ (mm^{-1}), rad. type	0.815, Cu $K\alpha$	0.810, Cu $K\alpha$	0.809, Cu $K\alpha$
F_{000}	584	584	584
temp (K)	100(2)	100(2)	100(2)
Crystal form, color	plate, colorless	plate, colorless	plate, colorless
Crystal size, mm	0.31 x 0.09 x 0.05	0.26 x 0.19 x 0.06	0.33 x 0.09 x 0.08
Data collection			
Diffractometer	Bruker D8 Venture	Bruker D8 Venture	Bruker D8 Venture
T_{min}/T_{max}	0.6528/0.7536	0.6748/0.7536	0.6895/0.7536
No. of refls. (meas., uniq., and obs.)	27899/5318/5044	34533/5391/4911	27935/5392/5143
R_{int}	0.0359	0.0346	0.0323
θ_{max} (°)	72.223	72.187	72.175
Refinement			
R/R^2_{ω} (obs data)	0.0303/0.0739	0.0329/0.0802	0.0302/0.0765
R/R^2_{ω} (all data)	0.0327/0.0758	0.0379/0.0835	0.0322/0.0780
S	1.029	1.125	1.036
No. of refls.	5318	5391	5392
No. of parameters	392	392	382
$\Delta\rho_{max/min}$ (e·Å^{-3})	0.208/-0.198	0.217/-0.261	0.417/-0.182
Flack	0.11(5)	-0.04(8)	0.04(5)

Table S1. Crystallographic Data for the Diarylamide (**1**), Naphthylamide (**2**), and Benzoyl Phenylalanine (**3**) Systems. (Continued)

	(*S*)-**3**-2F/(*R*)-**3**-3F	(*S*)-**3**-2F/(*R*)-**3**-4F	(*R*)-**3**-3F/(*S*)-**3**-4F
Crystal data			
CCDC deposit no.	2442432	2442433	2442434
Empirical formula	$C_{32}H_{28}F_2N_2O_6$	$C_{32}H_{28}F_2N_2O_6$	$C_{32}H_{28}F_2N_2O_6$
Crystal System, space group	Monoclinic $C2$ (no. 5)	Monoclinic $P2_1$ (no. 4)	Monoclinic $P2_1$ (no. 4)
M_r	574.56	574.56	574.56
a, Å	21.7416(6)	11.1975(4)	11.2033(8)
b, Å	4.9510(1)	9.1928(3)	9.0791(7)
c, Å	25.6732(7)	14.0701(5)	14.3610(11)
α, deg	90	90	90
β, deg	90.285(2)	107.591(2)	107.805(4)
γ, deg	90	90	90
V, (Å^3)	2763.50(12)	1380.60(8)	1390.78(18)
Z, Z'	4, 1	2, 1	2, 1
D_{calc} (g cm^{-3})	1.381	1.382	1.372
μ (mm^{-1}), rad. type	0.877, Cu Kα	0.878, Cu Kα	0.872, Cu $K\alpha$
F_{000}	1200	600	600
temp (K)	100(2)	100(2)	100(2)
Crystal form, color	needle, colorless	needle, colorless	needle, colorless
Crystal size, mm	0.19x 0.05x 0.02	0.44 x 0.12 x 0.07	0.21 x 0.07 x 0.04
Data collection			
Diffractometer	Bruker D8 Venture	Bruker D8 Venture	Bruker D8 Venture
T_{min}/T_{max}	0.6724/0.7531	0.6322/0.7538	0.6472/0.7531
No. of refls. (meas., uniq., and obs.)	25121/5010/4663	40050/5554/5321	25496/5036/4751
R_{int}	0.0351	0.0402	0.0381
θ_{max} (°)	68.321	74.606	68.462
Refinement			
R/R^2_{ω} (obs data)	0.0344/0.0809	0.0392/0.1050	0.0398/0.1036
R/R^2_{ω} (all data)	0.0388/0.0853	0.0411/0.1073	0.0429/0.1063
S	1.063	1.032	1.052
No. of refls.	5010	5554	5036
No. of parameters	397	391	391
$\Delta\rho_{max/min}$ (e·Å^{-3})	0.223/-0.216	0.503/-0.337	0.719/-0.297
Flack	0.10(5)	0.17(5)	0.23(6)

Table S2. Hydrogen Bond Parameters for the Diarylamide (**1**), Naphthylamide (**2**), and Benzoyl Phenylalanine (**3**) systems.

Symmetry codes: (i) +*x*, 1+*y*, +*z*; (ii) 1+*x*, +*y*, +*z*; (iii) 2-*x*, -1.5+*y*, 1-*z*; (iv) 1-*x*, 1.5+*y*, 1-z; (v) -1+*x*, +*y*, +*z*; (vi) 0.5+*x*, 0.5-*y*, 0.5+*z*; (vii) *x*, -1+*y*, +*z*; (viii) 0.5+*x*, 0.5+*y*, +*z*; (ix) 1-*x*, 2-*y*, 1-z; (x) 1-*x*, -*y*, 1-*z*; (xi) 1+*x*, 0.5-*y*, 0.5+*z*; (xii) 1-*x*, 0.5+*y*, 1.5-*z*; (xiii) 1+*x*, 2+*y*, 1+*z*; (xiv) 1-*x*, -0.5+*y*, 1-*z*; (xv) 1-*x*, 0.5+*y*, 2-*z*; (xvi) 1-*x*, -0.5+*y*, 2-*z*; (xvii) 1-*x*, -0.5+*y*, -*z*.

Compound	D–H···A	D–H (Å)	H···A (Å)	D···A (Å)	D–H···A (°)
Diarylamide **1**					
(±)-**1**-2F	N1A-H1A···O1A^{i}	0.841(19)	2.467(19)	3.2122(17)	148.1(16)
	N1B-H1B···O1B^{i}	0.833(19)	2.410(19)	3.1689(17)	151.8(16)
(*S*)-**1**-2F/(*R*)-**1**-4F	N1A H1A···O1A^{ii}	0.88(4)	2.30(4)	3.146(3)	162(3)
	N1B H1B···O1B^{ii}	0.84(3)	2.36(3)	3.130(3)	153(2)
	N1B-H1B···F1D	0.84(3)	2.22(3)	2.746(7)	121(3)
(*S*)-**1**-3F/(*R*)-**1**-4F	N1A-H1A···O1B^{iii}	0.87(2)	2.11(2)	2.942(4)	161(3)
	N1B-H1B···O1A^{iv}	0.85(2)	2.12(2)	2.937(4)	162(3)
Naphthylamide **2**					
(±)-**2**-2F	N1A-H1A···O1A^{ii}	0.86(5)	2.09(5)	2.941(4)	175(4)
	N1B-H1B···O1B^{v}	0.86(6)	2.05(6)	2.906(4)	169(5)
	C4B-H4B···1A^{vi}	0.95	2.46	3.191(5)	133.8
(±)-**2**-3F	N1-H1···O1^{vii}	0.881(17)	2.088(17)	2.9638(15)	173.3(14)
	C9-H9B···F1^{viii}	0.98	2.45	3.137(13)	126.4
Benzoyl phenylalanine **3**					
(±)-**3**-2F	O2-H2···O3^{ix}	0.891(17)	1.732(18)	2.6234(19)	178(3)
	N1-H1···O1^{ii}	0.838(16)	2.316(17)	3.112(2)	159(2)
(±)-**3**-3F	O2-H2···O3^{x}	0.94(2)	1.69(2)	2.6288(15)	180(2)
	N1-H1···O1^{ii}	0.839(18)	2.230(18)	3.0389(16)	161.9(16)
	C10-H0A···F2^{xi}	0.99	2.27	3.104(4)	140.6
(±)-**3**-4F	O2-H2···O1^{xii}	0.896(17)	1.686(18)	2.5812(11)	177.9(16)
	N1-H1···O3^{xiii}	0.848(14)	2.086(15)	2.9125(12)	164.7(13)
(*S*)-**3**-H/(*R*)-**3**-2F	O2A-H2A···O1A^{xiv}	0.87(2)	1.73(2)	2.598(2)	175(3)
	N1A-H1A···O3B	0.87(2)	2.06(3)	2.886(3)	166(3)
	O2B-H2B···O1B^{xv}	0.89(2)	1.73(2)	2.596(3)	175(3)
	N1B-H1B···O3A	0.86(2)	2.15(2)	2.950(3)	154(3)
(*R*)-**3**-H/(*S*)-**3**-3F	O2A-H2A···O1A^{xvi}	0.89(2)	1.70(2)	2.590(3)	176(4)
	N1A-H1A···O3B	0.85(2)	2.05(2)	2.865(3)	160(3)
	O2B-H2B···O1B^{iv}	0.88(2)	1.72(2)	2.596(3)	173(4)
	N1B-H1B···O3A	0.87(2)	2.05(4)	2.909(3)	171(3)
(*S*)-**3**-H/(*R*)-**3**-4F	O2A-H2A···O1A^{iv}	0.89(2)	1.69(2)	2.578(2)	178(3)

	N1A-H1A···O3B	0.87(2)	2.04(2)	2.910(2)	172(3)
	O2B-H2B···O1B^{xvii}	0.88(2)	1.70(2)	2.580(2)	174(3)
	N1B-H1B···O3A	0.85(2)	2.08(2)	2.898(3)	161(3)
(*S*)-**3**-2F/(R)-**3**-3F	O2A-H2A···O3B	0.85(3)	1.78(3)	2.626(3)	169(8)
	O3A-H3A···O2B	0.84(3)	1.79(3)	2.630(3)	178(9)
	N1A-H1A···O1A^{i}	0.83(2)	2.13(2)	2.917(3)	160(3)
	O2B-H2B···O3A	0.85(3)	1.78(3)	2.630(3)	176(8)
	O3B-H3B···O2A	0.86(3)	1.77(3)	2.626(3)	171(8)
	N1B-H1B···O1B^{vii}	0.86(2)	2.05(2)	2.892(3)	165(3)
(*S*)-**3**-2F/(*R*)-**3**-4F	O2A-H2A···O1A^{xiv}	0.83(5)	1.78(5)	2.601(3)	168(4)
	N1A-H1A···O3B	0.82(3)	2.19(4)	2.944(3)	153(3)
	O2B-H2B···O1B^{iv}	0.92(4)	1.67(4)	2.588(3)	173(4)
	N1B-H1B···O3A	0.90(3)	2.00(3)	2.895(3)	171(3)
(*R*)-**3**-3F/(*S*)-**3**-4F	O2A-H2A···O1A^{xvii}	0.85(5)	1.76(5)	2.590(3)	167(5)
	N1A-H1A···O3B	0.83(4)	2.07(4)	2.872(4)	165(4)
	O2B-H2B···O1B^{iv}	0.96(5)	1.64(5)	2.602(3)	179(4)
	N1B-H1B···O3A	0.91(4)	2.04(4)	2.927(3)	164(3)

References

(1) Craddock, D. E.; Parks, M. J.; Taylor, L. A.; Wagner, B. L.; Ruf, M.; Wheeler, K. A. Increasing the Structural Boundary of Quasiracemate Formation: 4-Substituted Naphthylamides. *CrystEngComm* **2021**, *23* (1), 210–215. DOI: 10.1039/D0CE01331E.

(2) Koch, K. N.; Teo, A. J.; Wheeler, K. A. Dual Space Divergence in Small-Molecule Quasiracemates: Benzoyl Leucine and Phenylalanine Assemblies. *Chem. Commun.* **2024**, *60*, 2800–2803. DOI: 10.1039/D3CC06212K.

(3) Brandt, A. K.; Boyle, D. J.; Butler, J. P.; Gillingham, A. R.; Penner, S. E.; Spaniol, J. M.; Stockdill, A. K.; Vanderwall, M. M.; Yeraly, A.; Schepens, D. R.; Wheeler, K. A. Molecular Recognition and Shape Studies of 3- and 4-Substituted Diarylamide Quasiracemates. *Crystals* **2021**, *11* (12), 1596. DOI: 10.3390/cryst11121596.

(4) Tinsley, I. C.; Spaniol, J. M.; Wheeler, K. A. Mapping the Structural Boundaries of Quasiracemate Fractional Crystallization Using 2-Substituted Diarylamides. *Chem. Commun.* **2017**, *53* (33), 4601–4604. DOI: 10.1039/C7CC01638G.
